# Lip Squamous Cell Carcinoma: A Conceptual Framework for Integrating Molecular, Immune and Microenvironmental Drivers

**DOI:** 10.3390/ijms27219528

**Published:** 2026-10-25

**Authors:** Ylli Alicka, Skender Topi, Elona Gaxhja, Gianluca Baldini, Gino Orsini, Davide Pietropaoli, Maria Grazia Cifone, Maurizio Giuliani, Serena Altamura

**Affiliations:** 1 Faculty of Medical Technical Sciences, University of Elbasan “Aleksandër Xhuvani’”, 3001 Elbasan, Albania; ylli.alicka@uniel.edu.al (Y.A.); skender.topi@uniel.edu.al (S.T.); elona.gaxhja@uniel.edu.al (E.G.); 2 Department of Life, Health & Environment Sciences, University of L’Aquila, 67100 L’Aquila, Italy; gianluca.baldini@graduate.univaq.it (G.B.); gino.orsini@univaq.it (G.O.); davide.pietropaoli@univaq.it (D.P.); mariagrazia.cifone@univaq.it (M.G.C.); maurizio.giuliani@univaq.it (M.G.); 3 Unit of Plastic and Reconstructive Surgery, Casa di Cura “DI LORENZO”, 67051 Avezzano, Italy; 4 Department of Physical & Chemical Sciences, University of L’Aquila, 67100 L’Aquila, Italy

**Keywords:** lip squamous cell carcinoma, ultraviolet radiation, conceptual framework, molecular pathogenesis, epigenetic reprogramming, tumor microenvironment, immune surveillance, microbiome–immune–metabolic axis, biomarkers, precision oncology

## Abstract

Lip squamous cell carcinoma (LSCC) is a distinct subtype of head and neck squamous cell carcinoma strongly associated with ultraviolet (UV) radiation exposure and characterized by unique molecular, immune, and microenvironmental features. Although UV-induced photodamage represents the principal initiating event, increasing evidence indicates that LSCC progression results from complex interactions among genetic and epigenetic alterations, dysregulated signaling pathways, chronic inflammation, immune dysregulation, host–microbiome dynamics, and tumor microenvironment (TME) remodeling. Recent advances have expanded the traditional view of LSCC by highlighting the contributions of UV-induced DNA damage, tumor protein p53 (*TP53*) alterations, epigenetic reprogramming, aberrant activation of key signaling pathways, and progressive immune escape mechanisms. In parallel, growing attention has focused on the role of cancer-associated fibroblasts (CAFs), stromal remodeling, microbial dysbiosis, and disruption of the microbiome–immune–metabolic axis as important modulators of disease progression and biological heterogeneity. Emerging biomarkers, including microRNAs (miRNAs), long non-coding RNAs (lncRNAs), epigenetic signatures, liquid-biopsy analytes, immune-related markers, and microbiome-associated profiles, together with artificial intelligence (AI)-based analytical approaches, are creating new opportunities for diagnosis, risk stratification, disease monitoring, and precision oncology. Rather than providing a conventional descriptive overview, this review proposes an integrated conceptual framework in which molecular, immune, microbial, and microenvironmental drivers interact through continuous bidirectional crosstalk to shape LSCC initiation, progression, therapeutic responsiveness, and clinical heterogeneity. This system-oriented perspective may provide a foundation for future biomarker-guided and biologically informed approaches to LSCC management.

## 1. Introduction

Cancers of the lip and oral cavity represent a major global health burden, accounting for hundreds of thousands of new cases annually, with squamous cell carcinoma constituting the predominant histological subtype. Despite advances in prevention, diagnosis, and treatment, these malignancies continue to significantly impact morbidity, quality of life, and cancer-related mortality worldwide [1,2].

Lip squamous cell carcinoma (LSCC) represents a distinct subset of head and neck squamous cell carcinomas. Although traditionally classified among oral cavity malignancies, LSCC exhibits unique epidemiological, etiological, and biological characteristics that distinguish it from both conventional oral squamous cell carcinoma (OSCC) and cutaneous squamous cell carcinoma (cSCC). In particular, the prominent role of chronic ultraviolet (UV) radiation exposure in LSCC pathogenesis confers molecular and pathological features that partially overlap with those observed in UV-associated skin cancers while maintaining important site-specific characteristics [1].

Historically, LSCC has been investigated primarily from epidemiological, clinicopathological, and surgical perspectives. However, increasing evidence indicates that carcinogenesis is governed by complex interactions among environmental exposures, molecular alterations, immune regulation, chronic inflammation, host–microbiome dynamics, and tumor microenvironment (TME) remodeling. Consequently, LSCC is increasingly recognized as a biologically heterogeneous disease whose behavior cannot be fully explained by conventional clinicopathological parameters alone [3,4,5,6].

Recent advances in molecular pathology have expanded understanding of the mechanisms underlying LSCC development. UV-induced genomic instability, genetic and epigenetic alterations, dysregulated signaling pathways, and transcriptional reprogramming collectively contribute to malignant transformation and disease progression. These processes are increasingly recognized as interconnected biological events rather than isolated mechanisms, contributing to tumor heterogeneity and cellular adaptation [4,7,8,9].

Increasing attention has also been devoted to immunopathological mechanisms and the role of the TME. Alterations in antigen presentation, immune surveillance, immune-cell functionality, and stromal remodeling contribute to the establishment of an immunosuppressive microenvironment capable of sustaining tumor growth and promoting immune escape. In particular, dysfunction of Langerhans cells, immune checkpoint activation, and cancer-associated fibroblast (CAF)-mediated crosstalk have emerged as important determinants of disease behavior and therapeutic responsiveness [5,6,10,11].

In parallel, growing evidence supports a biologically relevant role for the oral microbiome in carcinogenesis. Microbial dysbiosis has been associated with chronic inflammation, epithelial barrier dysfunction, immune dysregulation, and activation of tumor-promoting pathways. These findings have led to the emergence of the microbiome–immune–metabolic axis as a conceptual framework linking microbial communities, immune responses, epithelial homeostasis, and metabolic regulation in disease progression [12,13,14].

Recent studies have further highlighted the importance of oral barrier immunometabolism, a complex regulatory network integrating epithelial barrier function, resident microbiota, immune responses, and metabolic pathways. Disruption of this system may contribute to persistent inflammatory activation and dysregulated host–microbe interactions, thereby creating biological conditions that favor carcinogenesis [15]. The growing recognition of the microbiome–immune–metabolic axis has also stimulated interest in microbiome-targeted therapeutic strategies, although their role in LSCC remains largely investigational [16,17].

Advances in molecular and translational oncology have further expanded interest in biomarker discovery. Molecular, epigenetic, immune-related, microbiome-associated, and liquid-biopsy biomarkers are increasingly being investigated for their potential roles in disease assessment, patient stratification, and therapeutic guidance. At the same time, artificial intelligence (AI)-based approaches are emerging as promising tools for integrating molecular, pathological, and clinical information into increasingly sophisticated predictive frameworks [18,19,20].

Collectively, these advances support a contemporary view of LSCC as a multifactorial and biologically heterogeneous disease arising from continuous crosstalk among environmental exposures, molecular alterations, immune regulation, microbiome-associated mechanisms, and TME dynamics. Rather than acting as independent drivers, these processes form interconnected biological networks that collectively influence tumor initiation, progression, immune escape, biomarker expression, and therapeutic responsiveness. This evolving perspective has important implications not only for understanding carcinogenesis but also for the development of biologically informed and biomarker-driven approaches to disease management.

Rather than providing a conventional descriptive review of individual pathogenic mechanisms, this article proposes a conceptual framework integrating current evidence on UV-induced carcinogenesis, genetic and epigenetic alterations, dysregulated signaling pathways, immune dysregulation, TME remodeling, and microbiome-associated mechanisms in LSCC. By examining these interconnected biological determinants within a unified interpretative model, we aim to highlight emerging pathogenic networks, identify current knowledge gaps, and discuss future opportunities for biomarker development, translational research, and precision oncology.

### Literature Search Strategy

This review was developed through a narrative and concept-oriented analysis of the available literature on lip squamous cell carcinoma (LSCC) and related fields. Relevant studies were identified through searches of PubMed, Scopus, and Web of Science using combinations of keywords including “lip squamous cell carcinoma”, “actinic cheilitis”, “oral squamous cell carcinoma”, “cutaneous squamous cell carcinoma”, “ultraviolet radiation”, “tumor microenvironment”, “immune surveillance”, “cancer-associated fibroblasts”, “oral microbiome”, “epigenetics”, “biomarkers”, and “precision oncology”. Priority was given to original research articles, systematic reviews, meta-analyses, and recent high-quality reviews published in English. Given the limited availability of LSCC-specific data in several areas, evidence from biologically related entities, including oral squamous cell carcinoma and cutaneous squamous cell carcinoma, was also considered when relevant to support the development of an integrated conceptual framework of LSCC pathogenesis and progression.

## 2. Epidemiology

### 2.1. Global Burden and Geographic Distribution

Squamous cell carcinoma represents the predominant histological subtype of head and neck malignancies and accounts for the vast majority of cancers arising within the oral cavity and related anatomical sites [21]. Within this spectrum, LSCC constitutes a relatively uncommon but epidemiologically distinct entity that differs from conventional OSCC and cSCC in terms of etiopathogenesis and risk-factor profile [1].

Contemporary epidemiological evidence indicates that LSCC accounts for approximately 4–5% of malignancies arising within the lip, oral cavity, and related anatomical regions worldwide [22].

The geographic distribution of LSCC exhibits substantial variability, reflecting differences in cumulative UV exposure, occupational activities, environmental conditions, and population-specific susceptibility factors. Higher incidence rates have consistently been reported in regions characterized by intense sunlight exposure, including Southern Europe, Australia, and parts of South America, whereas lower rates are generally observed in Northern Europe and several Asian countries [22,23].

Despite regional differences in incidence, mortality remains disproportionately higher in low- and middle-income countries, where delayed diagnosis, limited access to specialized healthcare services, and inequalities in cancer management continue to represent major challenges [2]. These observations emphasize the importance of preventive strategies focused on UV protection, early recognition of potentially malignant lip lesions, and timely access to specialized care.

Importantly, the marked geographic heterogeneity of LSCC supports the notion that disease occurrence cannot be explained solely by environmental exposure. Rather, cumulative UV damage interacts with host-specific molecular alterations, immune responses, and microenvironmental factors that collectively influence disease initiation and progression [4,5,6]. Understanding these epidemiological patterns therefore provides an essential foundation for the integrated molecular, immune, and microenvironmental framework discussed in the following sections.

### 2.2. Demographic Characteristics and Sex-Related Considerations

LSCC predominantly affects older adults, with most diagnoses occurring after the sixth decade of life. This age distribution likely reflects the cumulative effects of long-term UV exposure together with the progressive accumulation of molecular and cellular alterations associated with carcinogenesis [1,24,25].

A marked male predominance has historically been reported across epidemiological studies, with male-to-female ratios generally ranging from approximately 2:1 to 4:1 [1]. This disparity has largely been attributed to differences in cumulative UV exposure, particularly among individuals engaged in outdoor occupations, as well as historically higher prevalence rates of tobacco and alcohol consumption among men in several populations [26,27].

Recent epidemiological evidence suggests that this sex gap may be narrowing in some geographic regions, potentially reflecting changes in occupational exposure patterns, lifestyle habits, and increased adoption of sun-protective behaviors [25,27]. The more widespread use of photoprotective cosmetic products has also been proposed as a contributing factor to the historically lower incidence observed among women.

Although hormonal, genetic, and immunological factors have been implicated in sex-related differences across several malignancies, evidence specifically addressing biological sex as a determinant of LSCC susceptibility and progression remains limited. Consequently, most currently observed epidemiological disparities appear to be related to environmental and behavioral factors rather than to well-established sex-specific biological mechanisms [1,27].

Overall, current evidence indicates that demographic patterns in LSCC are shaped primarily by cumulative environmental exposure. However, the observation that only a subset of exposed individuals ultimately develops cancer suggests that additional host-related factors may influence disease susceptibility. A better understanding of the molecular, immune, and microenvironmental determinants underlying individual vulnerability may help explain the variability observed across demographic groups and further refine current models of LSCC pathogenesis.

### 2.3. Anatomical Distribution

LSCC exhibits a marked predilection for the lower lip, which accounts for approximately 85–90% of cases reported in epidemiological series [1]. This anatomical distribution is widely attributed to the greater cumulative exposure of the lower lip to solar UV radiation compared with the upper lip, further supporting the central role of UV-associated carcinogenesis.

The lower lip represents a unique anatomical interface between the cutaneous and oral environments and is continuously exposed to external stimuli, including solar radiation, temperature fluctuations, mechanical trauma, and environmental stressors. This distinctive exposure profile may facilitate the accumulation of molecular damage, promote chronic inflammatory responses, and contribute to increased susceptibility to malignant transformation [1,28].

The predominance of LSCC in the lower lip further highlights its relationship with cSCC, as both entities share chronic UV exposure as a major etiological driver and exhibit several features associated with photodamage-induced carcinogenesis [3,29]. At the same time, the anatomical location of LSCC at the junction between cutaneous and oral tissues suggests the involvement of additional local factors, including tissue-specific immune surveillance mechanisms, epithelial barrier alterations, and microenvironmental interactions that may influence disease initiation and progression.

Although considerably less frequent, tumors arising from the upper lip may display distinct epidemiological characteristics and risk-factor profiles, potentially reflecting differences in local anatomy and patterns of environmental exposure.

Particular attention should also be devoted to lesions involving the lip commissure, a region characterized by complex anatomy and rich lymphatic drainage. Tumors arising in this area have been associated with increased risks of deep tissue infiltration, regional nodal metastasis, and local recurrence [30].

Overall, the anatomical distribution of LSCC reflects more than differential UV exposure alone. Rather, disease occurrence appears to result from the interaction between site-specific environmental insults, local tissue architecture, immune surveillance, and microenvironmental factors that collectively modulate susceptibility to malignant transformation and disease progression. Consequently, anatomical location may represent an important determinant within the broader biological framework underlying LSCC pathogenesis.

### 2.4. Temporal Trends and Epidemiological Challenges

In several high-income countries, the incidence of LSCC appears to be stable or modestly declining, a trend commonly attributed to increased awareness of sun-protective measures, implementation of preventive strategies, and reduced exposure to traditional behavioral risk factors [2,22,24].

The exposed location of the lip may facilitate earlier recognition of suspicious lesions compared with other oral malignancies, potentially contributing to improved clinical outcomes when diagnosis is established at an early stage [31,32]. Nevertheless, delayed diagnosis continues to occur in a subset of patients, particularly in settings characterized by limited access to specialized care and cancer surveillance programs.

Despite these encouraging observations, the epidemiological assessment of LSCC remains challenging. One of the major limitations arises from inconsistencies in disease classification and reporting practices. In many cancer registries, lip tumors continue to be grouped within broader oral cavity or head and neck cancer categories, limiting the availability of reliable subsite-specific estimates of incidence, prevalence, survival, and temporal trends [22].

Improved registry harmonization, standardized disease classification, and subsite-specific reporting are therefore essential to facilitate a more accurate understanding of disease burden, refine risk assessment, and support effective prevention and surveillance strategies. Integrating epidemiological observations with molecular, immune, and microenvironmental data may further enhance the identification of high-risk populations and provide deeper insights into disease susceptibility and progression.

Overall, continued efforts to standardize epidemiological reporting and incorporate biological characterization into population-based studies will be critical for advancing our understanding of LSCC and supporting the development of more precise disease models.

### 2.5. Additional Risk Factors

Although chronic UV radiation is widely regarded as the principal etiological driver of LSCC, a variety of behavioral, environmental, and host-related factors may contribute to disease susceptibility and progression.

Tobacco consumption remains one of the most consistently recognized co-factors. Exposure of the lip epithelium to tobacco-derived carcinogens may potentiate the effects of UV-induced damage, thereby promoting genomic instability and malignant transformation. While tobacco appears to play a less dominant role in LSCC than in conventional intraoral squamous cell carcinoma, its contribution to the overall carcinogenic process should not be overlooked [1,27].

The independent role of alcohol consumption remains less clearly established. Current evidence suggests that alcohol may function primarily as a modifying factor, particularly when combined with tobacco exposure and other carcinogenic influences [27].

Immunosuppression represents another important risk modifier. Organ transplant recipients, individuals receiving long-term immunosuppressive therapy, and patients with impaired immune surveillance exhibit increased susceptibility to squamous cell carcinomas, including tumors arising on the lip [1,5]. These observations further support the importance of immune competence in limiting malignant transformation and controlling tumor development.

Several host-related characteristics have also been associated with increased risk, including advanced age, fair skin phenotype, chronic actinic damage, and prolonged cumulative sun exposure. By contrast, the contribution of chronic local irritation, poor oral hygiene, and inherited genetic predisposition remains less clearly established [1,27].

Overall, available evidence supports the concept that LSCC is predominantly an exposure-driven malignancy in which chronic UV radiation acts as the principal carcinogenic stimulus. However, disease risk and progression appear to be shaped by a complex interplay of behavioral exposures, host-related characteristics, immune competence, and biological susceptibility factors. These observations reinforce the view that LSCC development cannot be attributed to a single etiological determinant but rather results from the interaction of environmental, molecular, immune, and microenvironmental influences that collectively contribute to carcinogenesis.

Given the unique anatomical and biological characteristics of LSCC, a comparative overview of its major similarities and differences with OSCC and cSCC is provided in Table 1.

## 3. Molecular and Cellular Mechanisms of LSCC Carcinogenesis

### 3.1. UV Radiation-Induced Carcinogenesis

Among the environmental factors implicated in lip carcinogenesis, chronic solar UV radiation exposure is widely recognized as the predominant etiological driver of LSCC, particularly in tumors arising from the lower lip. Owing to its anatomical position, the lower lip is continuously exposed to solar radiation and therefore accumulates UV-induced molecular damage over time. This distinctive exposure profile contributes to the unique biological features of LSCC, a malignancy that shares pathogenetic characteristics with both OSCC and cSCC [1].

**Figure 1:**
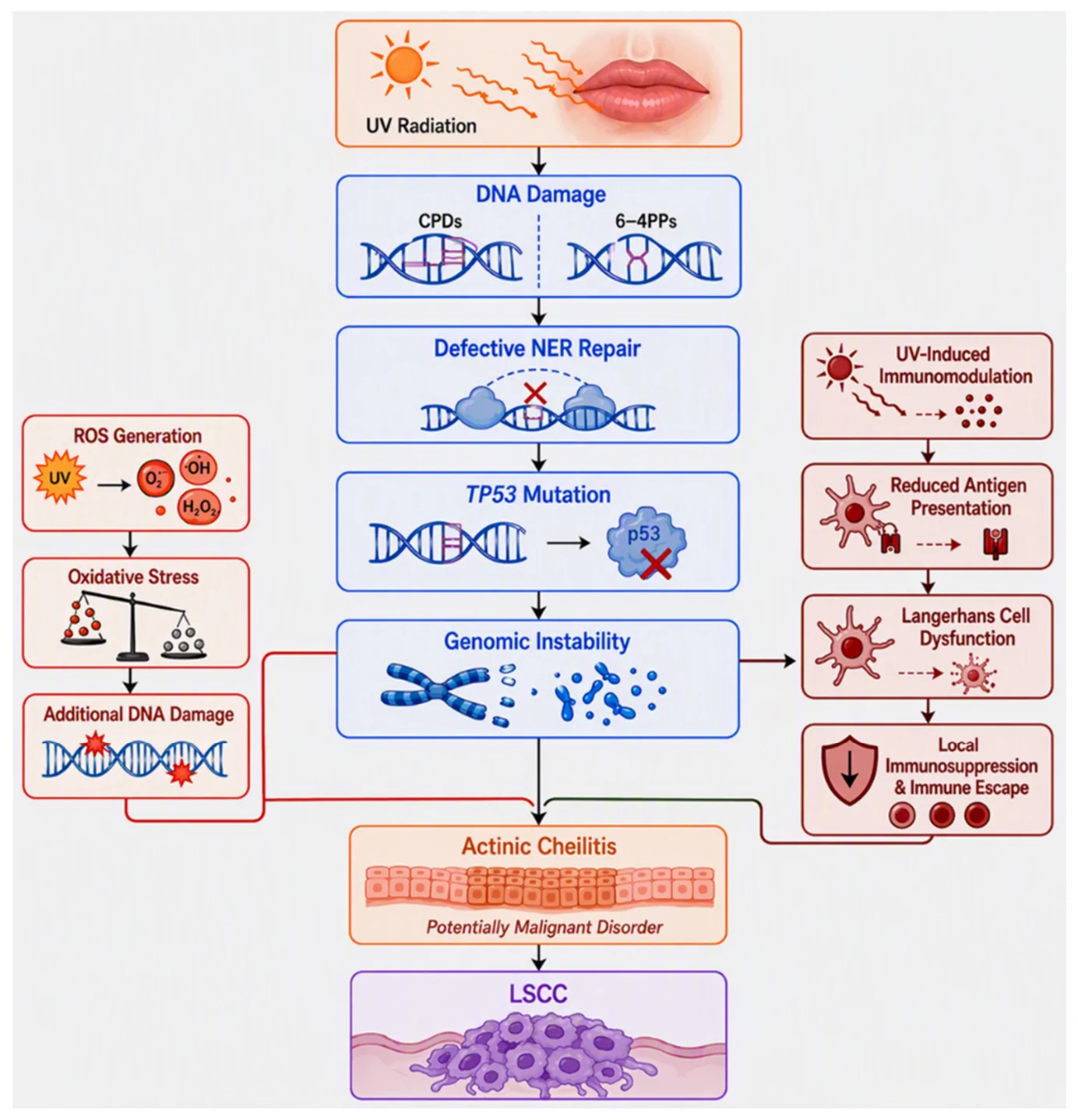
UV radiation-induced carcinogenesis in LSCC. Chronic UV radiation promotes carcinogenesis through the induction of DNA photolesions, including CPDs and 6–4PPs. Defective NER facilitates the accumulation of *TP53* mutations and genomic instability. Concurrently, UV-induced ROS contribute to oxidative stress and secondary DNA damage, while immunomodulatory effects impair antigen presentation, promote Langerhans cell dysfunction, and facilitate local immunosuppression. Together, these interconnected molecular and immunological mechanisms drive the transition from actinic cheilitis to invasive LSCC and establish the biological foundation for subsequent genetic, epigenetic, immune, and microenvironmental alterations. Colors are used to distinguish the major pathogenic mechanisms involved in UV-induced carcinogenesis, while arrows indicate the direction of biological events and disease progression. The red cross symbol denotes defective or impaired biological functions.

The carcinogenic effects of UV radiation extend far beyond its direct mutagenic potential. Ultraviolet B (UVB) radiation induces DNA photolesions, primarily cyclobutane pyrimidine dimers (CPDs) and pyrimidine-(6–4)-pyrimidone photoproducts (6–4PPs), which interfere with DNA replication and transcription (Figure 1). When these lesions escape repair by nucleotide excision repair (NER) mechanisms, they promote the accumulation of somatic mutations and contribute to genomic instability, one of the fundamental hallmarks of cancer [26,33,34].

One of the best-characterized molecular consequences of chronic UV exposure is the development of tumor protein p53 (*TP53*) mutations. UV-signature *TP53* alterations represent early events in squamous carcinogenesis and constitute one of the most recognizable molecular fingerprints of UV-induced malignancies. Through its pivotal role in regulating cell-cycle arrest, DNA repair, senescence, and apoptosis, p53 functions as a critical guardian of genomic integrity. Consequently, loss of p53 activity facilitates the clonal expansion of genetically damaged keratinocytes and promotes malignant transformation [3,7,8].

Beyond direct DNA injury, UV radiation contributes to carcinogenesis through the generation of reactive oxygen species (ROS) and the establishment of chronic oxidative stress. These processes may induce lipid peroxidation, protein oxidation, mitochondrial dysfunction, and secondary DNA damage, thereby amplifying genomic instability and promoting activation of signaling pathways involved in cellular proliferation, survival, inflammation, and adaptation to environmental stress [4,33]. Importantly, oxidative stress represents a key biological link between environmental exposure and molecular reprogramming that characterizes malignant progression.

Increasing evidence also indicates that chronic UV exposure exerts profound immunomodulatory effects within the epithelial microenvironment. UV radiation can impair antigen presentation, alter cytokine production, reduce immune surveillance, and modulate the activity of dendritic cells, including Langerhans cells. Collectively, these alterations contribute to local immunosuppression and facilitate tumor immune escape, thereby creating conditions that favor disease initiation and progression [5,35]. These observations further support the concept that UV radiation contributes not only to genomic damage but also to progressive remodeling of the local immune microenvironment.

The biological significance of chronic UV exposure is clinically exemplified by actinic cheilitis, the most common potentially malignant disorder of the lip. Histopathologically characterized by varying degrees of epithelial dysplasia, actinic cheilitis is widely regarded as the principal precursor lesion of LSCC and represents a continuum between chronic photodamage and invasive carcinoma. Recent evidence continues to support its malignant transformation potential, highlighting the importance of early diagnosis, risk stratification, and surveillance of high-risk lesions [29].

Collectively, current evidence indicates that the role of UV radiation in LSCC extends beyond simple carcinogen exposure. Chronic UV damage initiates a cascade of interconnected events involving genomic instability, oxidative stress, immune dysregulation, and progressive microenvironmental remodeling. Within this framework, UV radiation acts as the initiating driver of a broader pathogenic network in which molecular, immune, and tissue-level alterations interact to promote tumor initiation, progression, and biological heterogeneity. Understanding these interconnected processes provides the foundation for the integrated conceptual model of LSCC discussed in the following sections.

### 3.2. Genetic and Epigenetic Alterations

Although the molecular landscape of LSCC remains less extensively characterized than that of OSCC and cSCC, available evidence suggests that LSCC development results from the progressive interaction between UV-induced genomic damage, genetic susceptibility, and epigenetic reprogramming. Together, these processes drive malignant transformation and contribute to the biological heterogeneity of the disease [3,4].

Building upon UV-induced genomic damage, *TP53* alterations represent one of the earliest and most extensively characterized molecular events associated with LSCC carcinogenesis. UV-induced *TP53* mutations compromise multiple cellular defense mechanisms, including DNA repair, cell-cycle regulation, apoptosis, and senescence, thereby facilitating the clonal expansion of genetically damaged keratinocytes. These alterations contribute to genomic instability and represent a hallmark of UV-associated squamous carcinogenesis [3,7,8]. Beyond their role as early molecular events, *TP53* abnormalities may also influence immune regulation, cellular adaptation to environmental stress, and interactions with the surrounding microenvironment, highlighting their central position within the pathogenic network of LSCC.

Beyond *TP53*, evidence derived primarily from OSCC and cSCC indicates that alterations affecting genes involved in epithelial differentiation, chromatin organization, apoptosis, and genome maintenance may contribute to disease progression [3,4]. Although direct LSCC-specific evidence remains limited, available data support the concept that progressive disruption of these regulatory pathways contributes to the transition from actinic cheilitis to invasive carcinoma. These observations further suggest that LSCC shares fundamental molecular mechanisms with other squamous malignancies while maintaining distinct biological characteristics related to its unique anatomical location and etiological profile.

**Figure 2:**
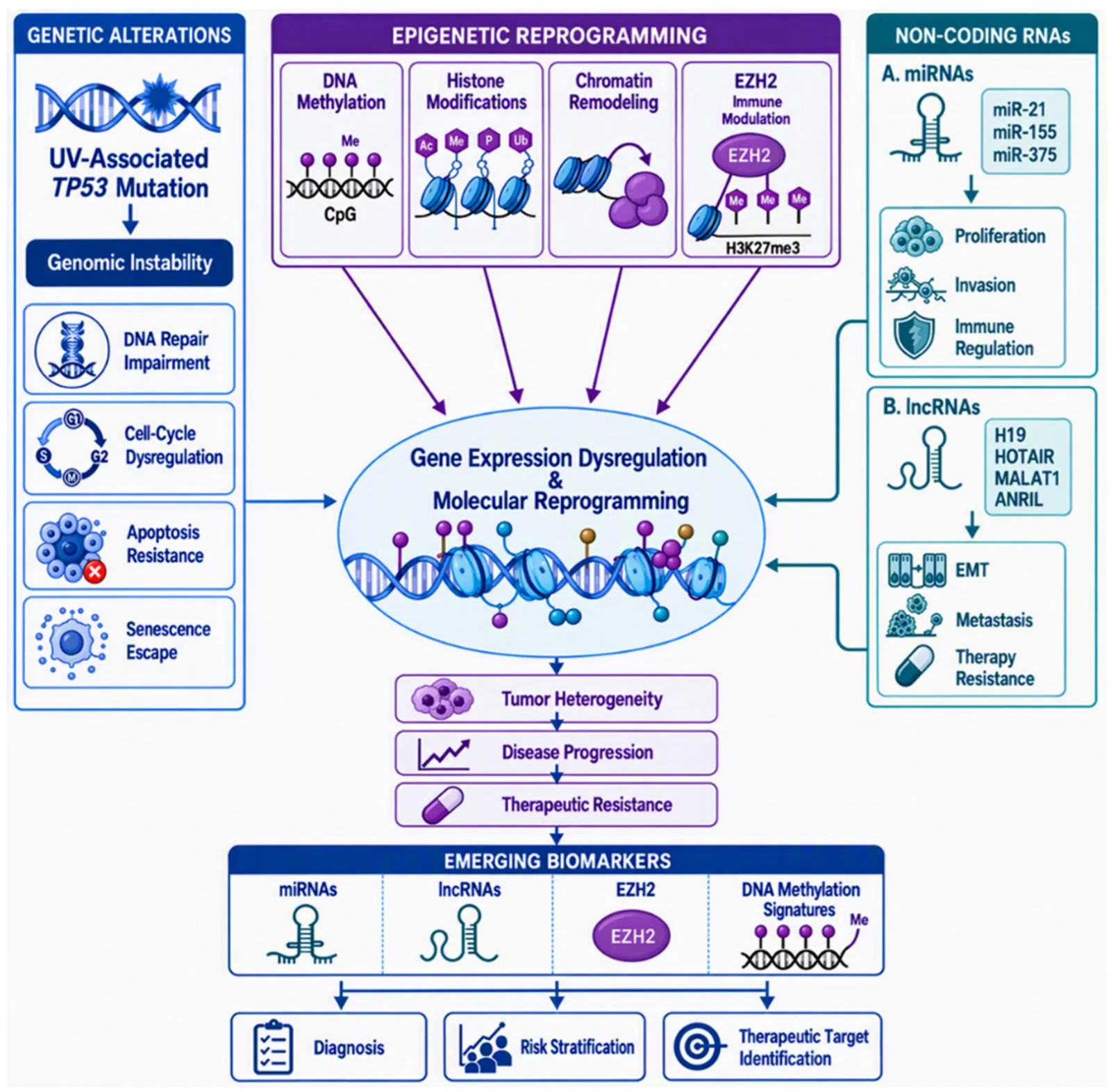
Genetic and epigenetic alterations involved in LSCC carcinogenesis. UV-associated *TP53* mutations and the resulting genomic instability cooperate with epigenetic reprogramming mechanisms, including DNA methylation, histone modifications, chromatin remodeling, non-coding RNA dysregulation, and EZH2-mediated transcriptional regulation, leading to widespread gene-expression dysregulation. These interconnected molecular events contribute to tumor heterogeneity, disease progression, therapeutic resistance, and adaptation to immune and microenvironmental pressures. In parallel, they support the emergence of clinically relevant biomarkers, including miRNAs, lncRNAs, EZH2, and DNA methylation signatures. Collectively, these biological alterations represent a central molecular hub linking environmental exposure to the immune and microenvironmental drivers of LSCC and provide opportunities for diagnosis, risk stratification, biomarker development, and precision oncology approaches. Colors are used to distinguish the major molecular mechanisms involved in LSCC carcinogenesis, while arrows indicate the direction of regulatory relationships and downstream biological effects.

In parallel with genetic abnormalities, epigenetic dysregulation has emerged as a critical component of squamous carcinogenesis (Figure 2). Epigenetic modifications can influence gene expression without altering the underlying DNA sequence and include aberrant DNA methylation, histone modifications, chromatin remodeling, and dysregulated expression of non-coding RNAs. Importantly, epigenetic mechanisms may provide a biological bridge between environmental exposures and long-term transcriptional reprogramming, thereby influencing cellular responses to UV-induced damage and microenvironmental stimuli. These processes are increasingly recognized as important mediators of tumor initiation, progression, immune evasion, therapeutic resistance, and biological heterogeneity in head and neck malignancies, while also providing a biological basis for subsequent biomarker development and therapeutic stratification strategies [9,19].

Aberrant DNA methylation of tumor suppressor genes has been associated with transcriptional silencing and disruption of cellular homeostasis, whereas altered histone modifications may profoundly affect chromatin accessibility and gene-expression programs involved in proliferation, differentiation, immune regulation, and cellular adaptation to environmental stress. These epigenetic alterations contribute to tumor heterogeneity and may facilitate adaptation to microenvironmental pressures, further supporting their potential relevance as early biomarkers of oral carcinogenesis [9,36].

Non-coding RNAs represent an additional layer of epigenetic regulation. While most available evidence derives from OSCC and related squamous malignancies, several microRNAs (miRNAs), including miR-21, miR-155, and miR-375, have been implicated in pathways controlling cell proliferation, apoptosis, invasion, and immune responses and are increasingly being investigated as candidate diagnostic and prognostic biomarkers potentially relevant to LSCC biology [37]. Likewise, long non-coding RNAs (lncRNAs), including H19, HOTAIR, MALAT1, and ANRIL, have been implicated in the regulatory networks governing epithelial–mesenchymal transition, metastatic dissemination, treatment resistance, and tumor progression. Through their ability to modulate transcriptional programs and cellular plasticity, these molecules are increasingly recognized as contributors to the biological evolution of squamous malignancies and may participate in the adaptive processes underlying disease progression [38,39].

Among epigenetic regulators, Enhancer of Zeste Homolog 2 (EZH2), the catalytic component of Polycomb Repressive Complex 2 (PRC2), has emerged as an important mediator of epigenetic reprogramming in squamous carcinogenesis. Through histone H3 lysine 27 trimethylation (H3K27me3), EZH2 regulates chromatin accessibility and transcriptional activity, thereby influencing cellular proliferation, differentiation, survival, and adaptive responses to environmental stress. Beyond its role in chromatin remodeling, growing evidence suggests that EZH2 may participate in immune regulation and tumor–microenvironment interactions, supporting its involvement in the broader biological networks that govern tumor progression. Recent studies have further identified increased EZH2 expression in high-risk LSCC, highlighting its potential relevance in disease development and progression [40].

Collectively, current evidence supports the view that LSCC arises through the dynamic interplay of UV-induced genomic damage, genetic instability, and epigenetic reprogramming. Rather than acting as isolated events, these alterations participate in interconnected regulatory networks that influence tumor initiation, progression, immune surveillance, microenvironmental remodeling, and therapeutic responsiveness. Within this conceptual framework, genetic and epigenetic alterations constitute a central biological hub linking environmental exposure to the molecular, immune, and microenvironmental drivers that shape LSCC heterogeneity and clinical behavior. Furthermore, a deeper understanding of these molecular events may facilitate the identification of novel tissue, salivary, circulating, and immune-related biomarkers while supporting biomarker-guided risk stratification and future precision oncology approaches [5,19,20].

### 3.3. Dysregulated Signaling Pathways

Building upon the genetic and epigenetic alterations described above, LSCC progression is further sustained by dysregulation of multiple intracellular signaling pathways that collectively regulate cellular proliferation, differentiation, survival, inflammation, angiogenesis, metabolic adaptation, and immune responses. Although molecular studies specifically focused on LSCC remain limited, evidence derived from OSCC and cSCC indicates that aberrant activation of several oncogenic networks represents a fundamental mechanism driving disease progression and biological heterogeneity. Among these, epidermal growth factor receptor (EGFR), the phosphoinositide 3-kinase/protein kinase B/mammalian target of rapamycin (PI3K/AKT/mTOR), mitogen-activated protein kinase (MAPK), nuclear factor-kappa B (NF-κB), and signal transducer and activator of transcription 3 (STAT3) signaling pathways have emerged as central regulators of squamous carcinogenesis [3,4].

#### 3.3.1. EGFR Signalling

EGFR is one of the most extensively investigated receptor tyrosine kinases in head and neck squamous cell carcinomas. Activation of EGFR promotes cellular proliferation, migration, angiogenesis, and resistance to apoptosis through engagement of multiple downstream signaling cascades. EGFR overexpression has been reported in a substantial proportion of squamous cell carcinomas and is frequently associated with tumor aggressiveness, disease progression, and therapeutic resistance [4,6].

Beyond its direct effects on tumor-cell proliferation, EGFR signaling participates in broader regulatory networks involving inflammatory mediators, stromal components, and the TME. Although evidence specifically focused on LSCC remains limited, sustained EGFR activation is increasingly recognized as a contributor to tumor progression and a potentially relevant target for future biomarker-guided and molecularly targeted therapeutic strategies.

#### 3.3.2. PI3K/AKT/mTOR Axis

Among the principal downstream effectors of EGFR, the PI3K/AKT/mTOR pathway plays a pivotal role in regulating cellular growth, metabolism, protein synthesis, proliferation, and survival. As one of the most frequently dysregulated signaling networks in squamous cell carcinogenesis, aberrant activation of this pathway has been implicated in multiple stages of tumor development and has been associated with enhanced proliferative capacity, inhibition of apoptosis, angiogenesis, invasion, and therapeutic resistance [3,4]. Activation of the PI3K/AKT/mTOR axis may occur through several mechanisms, including upstream EGFR stimulation, alterations affecting key regulatory components of the pathway, and crosstalk with other oncogenic signaling networks. Once activated, this signaling cascade promotes cellular survival and growth by enhancing protein synthesis, metabolic activity, and resistance to stress-induced apoptosis, thereby facilitating tumor progression and adaptation to adverse conditions [3,4].

Beyond its role in sustaining tumor-cell proliferation, the PI3K/AKT/mTOR pathway has emerged as a critical regulator of metabolic reprogramming and cellular adaptation to microenvironmental stress. Persistent activation of this signaling network enables cancer cells to respond to hypoxia, nutrient deprivation, oxidative stress, and therapeutic pressure, thereby supporting disease progression and contributing to biological heterogeneity [4,6].

Increasing evidence also suggests that PI3K/AKT/mTOR signaling influences important aspects of tumor–microenvironment interactions. Through its effects on angiogenesis, immune regulation, inflammatory signaling, and stromal-cell communication, this pathway contributes to the establishment of a microenvironment that supports tumor growth and survival. Moreover, PI3K/AKT/mTOR activation has been implicated in mechanisms of immune escape and resistance to anticancer therapies, further highlighting its relevance in tumor evolution [6].

Although direct molecular data in LSCC remain limited, findings derived from OSCC and cSCC suggest that dysregulation of the PI3K/AKT/mTOR axis is likely to represent an important component of LSCC biology. Within the conceptual framework proposed in this review, this pathway may serve as a critical mediator linking UV-induced molecular alterations, metabolic adaptation, immune modulation, and microenvironmental remodeling. Consequently, the PI3K/AKT/mTOR axis represents not only a key determinant of tumor-cell survival but also an important integrative signaling hub connecting molecular, immune, and microenvironmental drivers of disease progression. Its central role in coordinating these processes supports ongoing efforts to evaluate components of this pathway as potential biomarkers and therapeutic targets in squamous cell carcinogenesis.

#### 3.3.3. MAPK Pathway

The MAPK pathway represents another major signaling network involved in the regulation of cellular proliferation, differentiation, survival, and responses to environmental stress. Functioning downstream of growth factor receptors such as EGFR, the MAPK cascade serves as a critical mediator of signal transduction between extracellular stimuli and transcriptional programs governing cell fate and adaptation [3,4].

Activation of the MAPK pathway occurs through a highly coordinated signaling cascade involving RAS, RAF, MEK, and ERK proteins, ultimately leading to the regulation of genes involved in cell-cycle progression, proliferation, differentiation, and survival. Under physiological conditions, MAPK signaling contributes to tissue homeostasis and adaptive cellular responses. However, persistent or dysregulated activation can promote uncontrolled growth, resistance to apoptosis, and acquisition of malignant phenotypes.

In squamous carcinogenesis, aberrant MAPK signaling has been associated with enhanced proliferative activity, increased invasive potential, and adaptation to environmental stressors. Furthermore, MAPK activation contributes to cellular responses to oxidative stress, inflammatory stimuli, and growth factor signaling, highlighting its role as a central mediator of tumor-cell plasticity and adaptation [3,4].

Beyond its effects on tumor-cell intrinsic behavior, the MAPK pathway participates in dynamic interactions with the TME. Activation of MAPK signaling can influence cytokine production, inflammatory responses, extracellular matrix remodeling, and angiogenesis, thereby contributing to the establishment of biological conditions that support tumor progression. Increasing evidence also suggests extensive crosstalk between MAPK and other oncogenic pathways, including EGFR, PI3K/AKT/mTOR, NF-κB, and STAT3 signaling networks, reinforcing its role within the broader regulatory circuitry of squamous carcinogenesis.

Although the specific contribution of MAPK signaling to LSCC remains incompletely characterized, findings derived from OSCC and cSCC strongly support its involvement in tumor progression and adaptation to a dynamically changing microenvironment [3,4]. Given the shared molecular and etiological features observed across squamous malignancies, MAPK dysregulation is likely to represent an important component of the signaling landscape underlying LSCC development.

Within the conceptual framework proposed in this review, the MAPK pathway may be viewed as a key integrative node linking UV-induced cellular stress, genetic and epigenetic alterations, inflammatory signaling, and microenvironmental adaptation. Through these interconnected functions, MAPK contributes to the biological plasticity and heterogeneity that characterize LSCC progression and may represent a promising target for future biomarker-driven and therapeutic strategies.

#### 3.3.4. NF-κB and STAT3 Signaling

The interplay between chronic inflammation and carcinogenesis is largely mediated by persistent activation of the transcription factors NF-κB and STAT3. These pathways regulate the expression of a broad spectrum of genes involved in cytokine production, immune regulation, cell proliferation, apoptosis resistance, angiogenesis, epithelial–mesenchymal transition (EMT), and tumor immune escape [4,9].

Under physiological conditions, NF-κB and STAT3 contribute to tissue homeostasis, immune responses, and adaptation to environmental stress. However, sustained activation of these transcriptional programs promotes a chronic pro-tumorigenic state characterized by persistent inflammatory signaling, enhanced cellular survival, and progressive disruption of normal regulatory mechanisms. As a result, both pathways are increasingly recognized as central mediators of squamous carcinogenesis.

Accumulating evidence suggests that NF-κB and STAT3 function as critical molecular hubs linking many of the biological processes discussed throughout this review, including UV-induced cellular damage, chronic inflammation, genetic and epigenetic alterations, immune dysregulation, microbiome-associated perturbations, and stromal remodeling [5,6,9]. Through these functions, they integrate inflammatory, immune, stress-response, and survival signals, thereby sustaining carcinogenic processes and facilitating long-term biological adaptation.

Activation of NF-κB and STAT3 further promotes the production of inflammatory mediators, including interleukin-6 (IL-6), tumor necrosis factor-alpha (TNF-α), and interleukin-1 beta (IL-1β), establishing self-reinforcing inflammatory circuits that perpetuate tumor-promoting signaling [6,9]. These cytokine networks not only support tumor-cell proliferation and survival but also influence immune-cell recruitment, stromal activation, extracellular matrix remodeling, and angiogenesis, thereby shaping the evolving TME.

Importantly, NF-κB and STAT3 exhibit extensive bidirectional crosstalk with other oncogenic pathways, including EGFR, PI3K/AKT/mTOR, and MAPK signaling. This coordinated network activity contributes to tumor plasticity, therapeutic resistance, and adaptation to microenvironmental pressures. Consequently, dysregulation of these transcription factors extends beyond inflammation alone and influences multiple dimensions of tumor biology.

Although direct studies in LSCC remain limited, evidence derived from OSCC and cSCC strongly supports a central role for NF-κB and STAT3 signaling in disease progression. Within the conceptual framework proposed in this review, these pathways may be viewed as key molecular integrators that connect environmental exposure, genomic instability, immune modulation, microbiome-associated signaling, and TME remodeling.

Collectively, dysregulation of EGFR, PI3K/AKT/mTOR, MAPK, NF-κB, and STAT3 signaling pathways represents a central component of LSCC molecular pathogenesis. Rather than functioning as isolated mechanisms, these pathways form highly interconnected regulatory networks that integrate genomic alterations, inflammatory responses, immune modulation, metabolic adaptation, and tumor–microenvironment interactions. Their extensive crosstalk contributes to disease heterogeneity and progression while providing a biological rationale for biomarker discovery, patient stratification, and the development of future molecularly targeted and immune-based therapeutic strategies [4,5,6].

### 3.4. TME and Immune Surveillance

**Figure 3:**
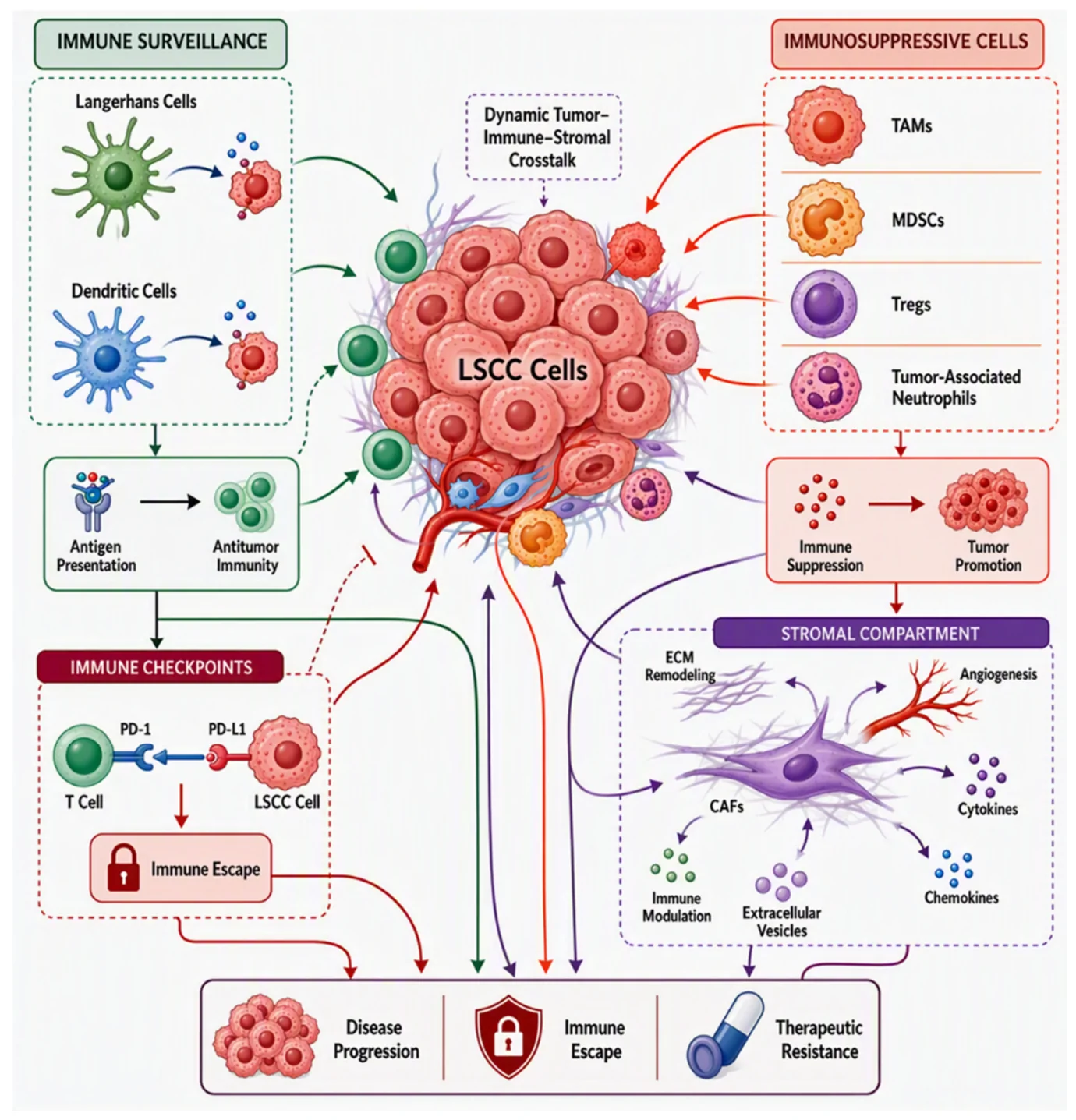
TME and immune surveillance in LSCC. Dynamic interactions among tumor cells, immune populations, immune checkpoint pathways, stromal components, and CAFs shape the biological behavior of LSCC. Progressive impairment of immune surveillance, activation of immunosuppressive cellular networks, PD-1/PD-L1 checkpoint signaling, and CAF-mediated stromal remodeling contribute to immune escape, extracellular matrix remodeling, angiogenesis, and therapeutic resistance. Together, these interconnected immune and microenvironmental mechanisms establish a dynamic tumor ecosystem that drives disease progression and influences clinical outcomes. Colors are used to distinguish the major cellular and stromal components of TME, while arrows indicate the direction of interaction and their contribution to LSCC progression, immune escape, and therapeutic resistance.

The contemporary understanding of squamous carcinogenesis extends beyond tumor-intrinsic molecular alterations and increasingly recognizes the pivotal role of the TME in shaping disease initiation, progression, and therapeutic responsiveness. In LSCC, as in other squamous cell carcinomas of the oral and maxillofacial region, carcinogenesis is orchestrated through dynamic interactions among transformed epithelial cells, immune populations, stromal fibroblasts, endothelial cells, extracellular matrix components, soluble mediators, and microbial-derived signals [5,6] (Figure 3).

Rather than representing a passive tissue compartment, the TME functions as a highly dynamic and adaptive ecosystem in which tumor cells continuously interact with surrounding cellular and acellular components. These interactions influence multiple biological processes, including proliferation, invasion, angiogenesis, metabolic reprogramming, immune surveillance, and therapeutic responsiveness, thereby contributing to disease heterogeneity and clinical behavior [5,6].

Accumulating evidence indicates that the composition and functional organization of the TME are critical determinants of tumor evolution [5,6]. Genetic and epigenetic alterations within tumor cells can reshape the surrounding microenvironment through the production of cytokines, growth factors, extracellular vesicles, and matrix-remodeling enzymes. In turn, stromal and immune components provide signals that support tumor-cell survival, adaptation, and progression. This bidirectional communication establishes a complex regulatory network linking molecular alterations to tissue-level biological responses. Consequently, the TME is increasingly viewed as a dynamic ecosystem that continuously interacts with tumor cells and contributes to disease heterogeneity and biological adaptation.

Importantly, many of the signaling pathways discussed in the previous sections, including EGFR, PI3K/AKT/mTOR, MAPK, NF-κB, and STAT3, exert substantial effects on the composition and function of the TME. Through these interconnected mechanisms, molecular dysregulation contributes to inflammatory signaling, immune modulation, angiogenesis, fibroblast activation, and extracellular matrix remodeling, thereby reinforcing tumor-promoting conditions.

Within the conceptual framework proposed in this review, the TME can therefore be viewed as the biological interface through which environmental exposures, molecular alterations, immune responses, and host-related factors converge to influence disease development and progression. Understanding these interactions is essential for explaining the biological heterogeneity of LSCC and may provide opportunities for biomarker discovery, risk stratification, and the development of novel therapeutic strategies.

#### 3.4.1. Immune Surveillance and Immunopathological Mechanisms

The immune compartment represents a central component of epithelial surveillance. Under physiological conditions, innate and adaptive immune cells continuously monitor epithelial integrity and contribute to the elimination of potentially transformed cells before malignant progression occurs (Figure 3). However, during carcinogenesis, neoplastic cells progressively acquire mechanisms capable of evading immune recognition and suppressing antitumor immunity, thereby favoring tumor persistence and expansion [4,5].

These observations are consistent with the concept of cancer immunoediting, whereby interactions between tumor cells and the immune system evolve through phases of elimination, equilibrium, and eventual immune escape. Although this model has not been extensively investigated in LSCC, evidence from OSCC and cSCC supports the concept that progressive impairment of immune surveillance may represent a critical event in squamous carcinogenesis [4,6].

Among the immune populations involved in oral epithelial homeostasis, dendritic cells and Langerhans cells play a particularly important role. Langerhans cells are specialized antigen-presenting cells located within the oral and lip epithelium, where they function as sentinels of mucosal immunity. Through antigen capture, processing, and presentation, these cells promote adaptive immune responses and facilitate early recognition of dysplastic and neoplastic cells.

A recent systematic review and network meta-analysis demonstrated reduced densities of Langerhans cells in LSCC compared with actinic cheilitis, supporting their potential involvement in malignant transformation and disease progression [10]. Furthermore, diminished Langerhans cell density has been associated with locoregional metastasis and impaired antitumor immune surveillance, highlighting their potential relevance as biomarkers of disease behavior.

#### 3.4.2. Immune Escape and Immune Checkpoint Signaling

Beyond dendritic-cell dysfunction, the LSCC microenvironment contains a diverse array of immune-cell populations capable of exerting both tumor-suppressive and tumor-promoting effects. Tumor-associated macrophages (TAMs), myeloid-derived suppressor cells (MDSCs), regulatory T lymphocytes (Tregs), tumor-associated neutrophils, and other inflammatory cell populations collectively contribute to the establishment of a highly heterogeneous immune landscape [5] (Figure 3).

Depending on their phenotypic and functional states, these immune populations may either support effective antitumor responses or promote immune evasion, tissue remodeling, angiogenesis, and disease progression. As carcinogenesis advances, the balance within the immune microenvironment often shifts toward immunosuppressive mechanisms that favor tumor persistence and adaptation. This transition reflects the dynamic interplay between tumor cells and immune populations and constitutes a critical component of cancer immunoediting.

Among the mechanisms contributing to immune escape, immune checkpoint signaling has emerged as a major regulator of antitumor immunity. In particular, the programmed cell death protein 1/programmed death-ligand 1 (PD-1/PD-L1) axis plays a central role in limiting T-cell activation and maintaining an immunosuppressive microenvironment. Persistent activation of this pathway may impair cytotoxic immune responses, facilitate tumor-cell survival, and contribute to disease progression [6]. Although LSCC-specific studies remain limited, evidence from OSCC and other squamous malignancies strongly supports the relevance of immune checkpoint signaling as a key mechanism of tumor immune evasion.

Chronic UV exposure may further amplify these processes through immunomodulatory effects that impair antigen presentation, alter cytokine networks, and promote local immunosuppression, thereby facilitating the persistence and expansion of genetically altered keratinocytes [5,35]. In this context, UV radiation contributes not only to the initiation of carcinogenesis through genomic damage but also to the establishment of immunological conditions that favor tumor survival and progression.

Increasing evidence further suggests that immune escape is closely interconnected with the molecular and signaling pathways discussed in previous sections. Activation of NF-κB, STAT3, and PI3K/AKT/mTOR signaling can promote the expression of immunosuppressive mediators, enhance recruitment of regulatory immune-cell populations, and support the maintenance of a tumor-permissive microenvironment. These interactions highlight the extensive crosstalk between molecular dysregulation and immune remodeling during disease evolution.

Within the conceptual framework proposed in this review, immune escape represents a pivotal biological transition linking UV-induced damage, molecular alterations, chronic inflammation, and TME remodeling. Rather than being a late and isolated event, immune evasion appears to evolve progressively throughout carcinogenesis and contributes significantly to disease heterogeneity, progression, and therapeutic responsiveness. A deeper understanding of these mechanisms may facilitate the identification of immune-related biomarkers and support the development of precision immunotherapeutic strategies for LSCC.

#### 3.4.3. Stromal Remodeling and CAFs

In recent years, increasing attention has been devoted to stromal components, particularly CAFs, as key regulators of tumor progression and microenvironmental remodeling. CAFs actively reshape the extracellular matrix (ECM), regulate tissue biomechanics, promote angiogenesis, and secrete a wide range of cytokines, chemokines, growth factors, and extracellular vesicles capable of influencing both tumor cells and immune populations [6,11]. Rather than functioning solely as structural support cells, CAFs are increasingly recognized as dynamic regulators of tumor ecology. Through reciprocal interactions with neoplastic cells, CAFs contribute to the establishment of a microenvironment that favors tumor growth, invasion, survival, and adaptation to biological stress. These effects are mediated not only through direct cell–cell interactions but also through extensive paracrine signaling networks that shape the composition and function of the surrounding tissue.

Recent studies have highlighted the remarkable heterogeneity of CAF populations, suggesting that distinct CAF subsets may exert diverse and sometimes context-dependent effects on tumor progression, prognosis, and therapeutic responsiveness [11]. This emerging complexity has challenged the traditional view of CAFs as a uniform stromal population and emphasizes their multifaceted role in squamous carcinogenesis. Importantly, several CAF-derived signaling pathways have been implicated in both stromal remodeling and immune regulation. Among the best-characterized are the IL-6–STAT3–PD-L1 and C-X-C motif chemokine ligand 12 (CXCL12)–C-X-C chemokine receptor type 4 (CXCR4) axes, which contribute to immune suppression, extracellular matrix remodeling, angiogenesis, and therapeutic resistance. These observations highlight the close interconnection between stromal activation and immune escape mechanisms within the TME [6,11].

Emerging evidence further indicates that CAFs actively participate in shaping the immune landscape of squamous carcinomas. Through macrophage polarization, recruitment of regulatory T cells (Tregs), modulation of immune checkpoint signaling, and regulation of inflammatory mediators, fibroblasts contribute to the establishment of an immunosuppressive niche capable of limiting effective antitumor immune responses [11]. Consequently, CAFs should be viewed not only as stromal regulators but also as important modulators of immune surveillance and tumor immune escape.

Within the conceptual framework proposed in this review, CAFs represent a critical biological interface linking molecular alterations, inflammatory signaling, immune regulation, and extracellular matrix remodeling. Their extensive interactions with tumor cells, immune populations, endothelial cells, and matrix components position them at the center of the dynamic crosstalk that drives disease progression and biological adaptation.

Taken together, these observations indicate that LSCC progression is governed not only by tumor-intrinsic molecular alterations but also by highly coordinated stromal and immunological processes occurring within a specialized TME. The continuous interaction among epithelial cells, CAFs, immune populations, extracellular matrix components, and soluble mediators creates a complex biological ecosystem that profoundly influences invasion, immune escape, therapeutic responsiveness, and disease evolution. A deeper understanding of these stromal–immune networks may facilitate the identification of novel microenvironment-related biomarkers and support the development of future stromal-targeted, immunomodulatory, and biomarker-guided therapeutic strategies [5,6,11].

### 3.5. Oral Microbiome and Chronic Inflammation

Over the past decade, growing evidence has highlighted the crucial role of the oral microbiome in maintaining mucosal homeostasis and regulating local immune responses. The oral cavity harbors one of the most diverse microbial ecosystems in the human body, comprising bacteria, fungi, viruses, and archaea that collectively contribute to epithelial integrity, immune surveillance, and metabolic regulation [12,13,15].

**Figure 4:**
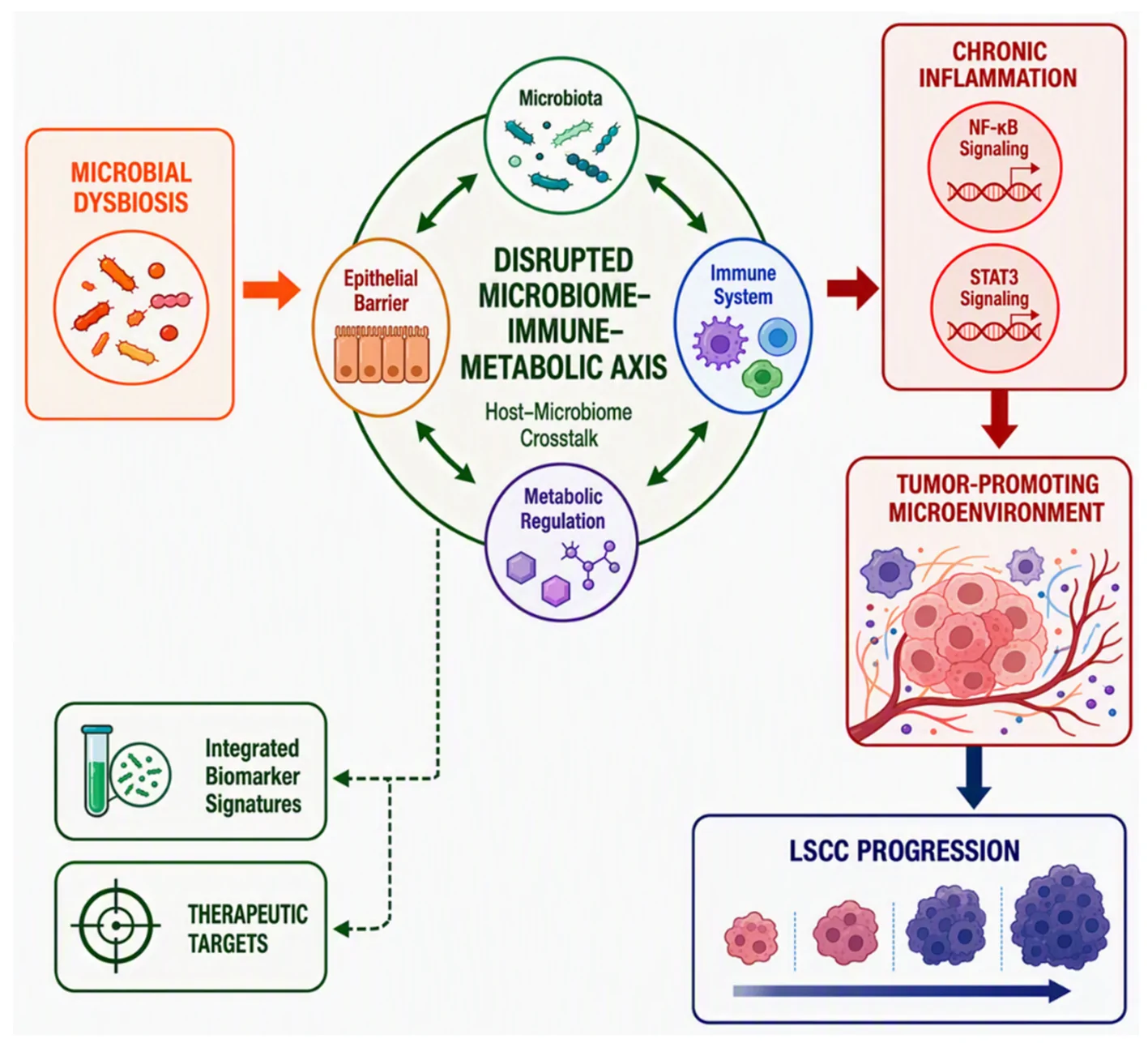
The microbiome–immune–metabolic axis in LSCC. Microbial dysbiosis disrupts the dynamic interactions among the oral microbiota, epithelial barrier, immune system, and metabolic regulatory networks, leading to chronic inflammation and activation of tumor-promoting signaling pathways, including NF-κB and STAT3. Through bidirectional interactions with the TME, these alterations contribute to immune dysregulation, microenvironmental remodeling, and LSCC progression. The microbiome–immune–metabolic axis therefore represents a potential source of integrated biomarkers and emerging therapeutic opportunities. Colors are used to distinguish the different biological components and pathways depicted in the figure, while arrows indicate the direction of interactions or disease progression.

Under physiological conditions, the oral microbiota exists in a dynamic state of equilibrium with the host, contributing to the maintenance of epithelial barrier integrity, immune homeostasis, and metabolic balance. Disruption of this finely regulated ecosystem, commonly referred to as dysbiosis, has increasingly been associated with oral carcinogenesis. Emerging evidence suggests that alterations in microbial communities may actively influence tumor initiation and progression through modulation of inflammatory responses, epithelial barrier function, and host immune regulation rather than representing merely secondary consequences of malignant transformation [12,14,41] (Figure 4).

Several microbial taxa have been associated with OSCC and potentially malignant oral disorders. In particular, enrichment of *Fusobacterium* spp. and *Porphyromonas* spp. has been consistently linked to tumor-promoting processes. These microorganisms may contribute to carcinogenesis through activation of inflammatory signaling pathways, modulation of host immune responses, disruption of epithelial barrier integrity, generation of bioactive metabolites, and promotion of a permissive TME [12,41,42]. Although direct evidence in LSCC remains limited, these observations suggest that microbial dysbiosis may represent an additional factor capable of influencing disease evolution within susceptible epithelial tissues.

The biological effects of dysbiosis appear to be closely interconnected with chronic inflammation. Persistent microbial stimulation can induce sustained production of pro-inflammatory mediators and activate signaling networks such as NF-κB and STAT3, which regulate cell proliferation, apoptosis resistance, angiogenesis, immune evasion, and tumor progression [13,41]. In this context, chronic inflammation may act as a mechanistic bridge linking microbial alterations to carcinogenic signaling pathways and progressive tissue remodeling.

Beyond conventional inflammatory pathways, increasing attention has been devoted to the concept of oral barrier immunometabolism, which describes the dynamic interactions among epithelial cells, resident microbiota, immune responses, and metabolic networks. Alterations in this integrated regulatory system may lead to persistent inflammatory activation, impaired epithelial resilience, dysregulated host–microbe interactions, and long-term metabolic reprogramming, thereby creating biological conditions favorable to carcinogenesis [15]. This emerging paradigm provides a broader interpretative framework through which environmental exposures, immune regulation, and microbial communities can be examined as interconnected determinants of disease susceptibility. Recent studies further suggest that microbial communities may influence carcinogenesis through modulation of oxidative stress responses, epithelial signaling pathways, local immune regulation, and production of metabolites capable of shaping the surrounding biological environment [14,43]. These observations reinforce the concept that the microbiome may participate in multiple layers of tumor biology extending beyond inflammation alone.

Importantly, interactions between the oral microbiome and the TME are increasingly recognized as bidirectional and highly dynamic. Microbial-derived metabolites and bacterial products can influence immune-cell recruitment, antigen presentation, cytokine production, stromal remodeling, and local metabolic pathways. Conversely, tumor-associated alterations in tissue architecture, oxygen availability, immune regulation, and inflammatory status may further modify microbial composition, generating self-reinforcing cycles of dysbiosis and chronic inflammation [5,13].

Although direct LSCC-specific evidence remains limited, the anatomical and biological continuity between the lip and oral mucosa supports the hypothesis that many microbiome-related mechanisms described in OSCC may also contribute to lip carcinogenesis. Nevertheless, dedicated studies are still required to define the specific microbial signatures and host–microbe interactions associated with LSCC.

Within the conceptual framework proposed in this review, the oral microbiome may be viewed as an active participant in a broader microbiome–immune–metabolic network linking dysbiosis, chronic inflammation, immune dysregulation, microenvironmental remodeling, and tumor progression. Rather than functioning as an isolated etiological factor, microbial dysbiosis appears to interact with UV-induced damage, molecular alterations, stromal remodeling, and host immune responses to influence disease evolution.

Collectively, current evidence indicates that the oral microbiome should no longer be regarded as a passive bystander in squamous carcinogenesis. Instead, microbial communities appear to participate in the regulation of epithelial homeostasis, inflammatory signaling, immune surveillance, metabolic adaptation, and tumor–microenvironment interactions. A deeper understanding of these processes may facilitate the identification of novel microbiome-related biomarkers and support the development of future preventive, diagnostic, and therapeutic strategies in LSCC.

### 3.6. Integrated Model of Carcinogenesis

Current evidence supports a multifactorial model of LSCC development in which chronic UV radiation acts as the principal initiating factor, while disease progression is driven by dynamic interactions among genetic and epigenetic alterations, dysregulated signaling pathways, immune responses, microbiome-associated mechanisms, chronic inflammation, and TME remodeling [4,6,7].

Rather than functioning as independent processes, these biological determinants form an interconnected pathogenic network that evolves throughout carcinogenesis. Chronic UV exposure induces genomic instability and promotes the accumulation of molecular alterations that facilitate malignant transformation. In parallel, epigenetic reprogramming amplifies transcriptional plasticity, contributes to tumor heterogeneity, and influences cellular adaptation to environmental and microenvironmental stressors [3,9,36].

At the molecular level, aberrant activation of signaling pathways including EGFR, PI3K/AKT/mTOR, MAPK, NF-κB, and STAT3 supports sustained proliferation, resistance to apoptosis, metabolic adaptation, and acquisition of aggressive phenotypic traits. Importantly, these pathways also establish extensive crosstalk with immune and stromal networks, thereby linking tumor-cell intrinsic alterations to broader biological processes occurring within the surrounding tissue environment [4,5,6].

As carcinogenesis progresses, TME increasingly becomes a central determinant of disease behavior. Alterations in immune surveillance, activation of immune checkpoint pathways, stromal remodeling, and CAF-mediated signaling collectively contribute to immune escape, angiogenesis, extracellular matrix remodeling, metastatic potential, and therapeutic resistance. These observations highlight the crucial role of immunopathological and stromal mechanisms in shaping disease evolution and clinical outcomes [5,6,10,11].

Concurrently, growing evidence suggests that microbial dysbiosis may further reinforce tumor-promoting conditions through modulation of inflammation, immune function, epithelial barrier integrity, and metabolic pathways. Within this emerging microbiome–immune–metabolic axis, bidirectional interactions among microbial communities, epithelial cells, immune populations, and local metabolic networks create additional layers of biological complexity that may influence individual susceptibility, disease progression, and biomarker profiles [12,14,15].

Collectively, these observations support a contemporary view of LSCC as a biologically heterogeneous disease arising from continuous crosstalk among environmental exposures, molecular alterations, immune responses, host–microbiome interactions, and microenvironmental remodeling. Within this conceptual framework, UV-induced damage serves as the initiating event, whereas tumor evolution is driven by the progressive integration of molecular, immune, microbial, and stromal processes that together determine disease behavior and clinical trajectory. This integrated model provides a biologically grounded framework for understanding LSCC pathogenesis and progression. Moreover, it offers a rationale for the development of multidimensional biomarker strategies and biologically informed therapeutic approaches capable of supporting future precision oncology initiatives [18,19,20].

**Figure 5:**
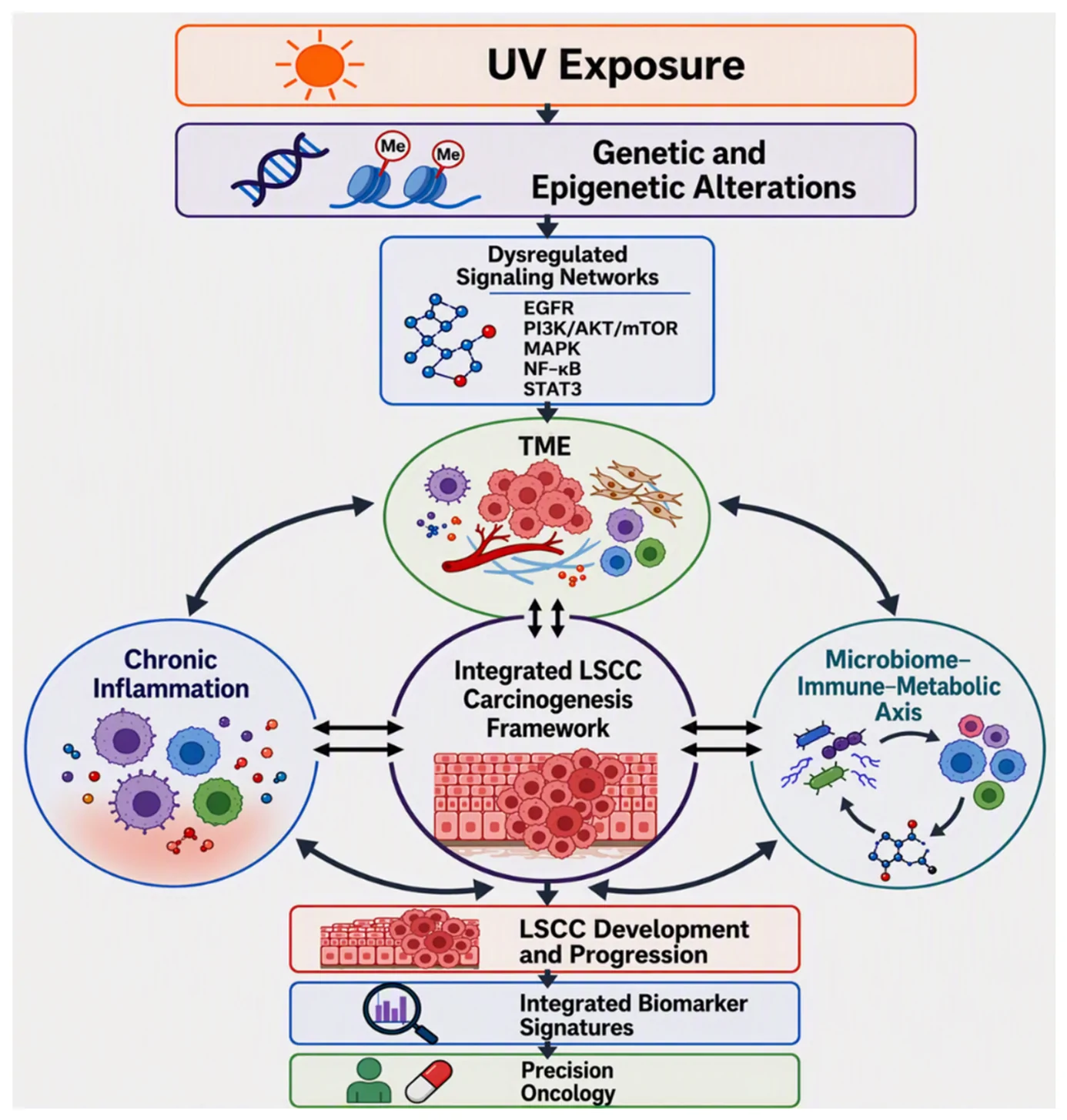
Integrated conceptual framework of LSCC carcinogenesis. Chronic UV exposure initiates a cascade of genetic and epigenetic alterations that promote dysregulation of key signaling networks and interact dynamically with chronic inflammation, immune responses, TME remodeling, and the microbiome–immune–metabolic axis. Through continuous bidirectional crosstalk, these molecular, immune, microbial, and microenvironmental drivers collectively shape disease initiation, progression, immune escape, biological heterogeneity, and therapeutic responsiveness. This integrated framework provides a biological basis for multidimensional biomarker development and future precision oncology approaches in LSCC. Colors are used to distinguish the major biological processes and components involved in LSCC carcinogenesis, while arrows indicate the direction of interactions, regulatory relationships, and disease progression. Symbols represent key cellular, molecular, and clinical features incorporated into the integrated framework.

An integrated overview of the major molecular, immunological, microbiological, and microenvironmental mechanisms involved in LSCC carcinogenesis is presented in Figure 5.

## 4. Emerging Biomarkers and Future Perspectives

### 4.1. Molecular and Epigenetic Biomarkers

The growing understanding of LSCC biology has stimulated increasing interest in the identification of biomarkers capable of improving diagnosis, risk stratification, treatment selection, and longitudinal disease monitoring. While current clinical management continues to rely largely on conventional clinicopathological parameters, advances in molecular biology have revealed a broad spectrum of candidate biomarkers that may provide additional insights into tumor behavior, biological heterogeneity, and therapeutic responsiveness [19].

Within the conceptual framework described in this review, molecular biomarkers are increasingly viewed as measurable manifestations of the complex biological networks underlying carcinogenesis. Genetic alterations, epigenetic reprogramming, immune modulation, and microenvironmental remodeling may each generate distinct biomarker signatures capable of reflecting disease initiation, progression, and response to therapy.

Among the most extensively investigated biomarker classes are miRNAs. Owing to their relative stability in biological fluids and accessibility through minimally invasive sampling procedures, several miRNAs, including miR-21, miR-155, and miR-375, have emerged as promising candidates for diagnostic, prognostic, and longitudinal monitoring applications in oral squamous malignancies [37]. Although most available evidence derives from OSCC and related squamous carcinomas, these molecules may also hold potential relevance for LSCC and warrant further investigation in disease-specific cohorts.

Similarly, lncRNAs have attracted growing interest as candidate molecular biomarkers because of their detectability in both tissue and circulating samples. Molecules such as H19, HOTAIR, MALAT1, and ANRIL may provide clinically relevant information regarding disease behavior, patient stratification, and therapeutic decision-making. Their potential integration into minimally invasive diagnostic and monitoring approaches further supports their emerging relevance within future biomarker-guided precision oncology frameworks [38,39].

Epigenetic biomarkers represent another rapidly evolving area of research. Aberrant DNA methylation signatures, chromatin remodeling patterns, and histone modifications have all been associated with tumor initiation, progression, and biological adaptation. Importantly, these alterations may capture early molecular events that precede overt malignant transformation, making them particularly attractive candidates for risk assessment and early detection strategies [9,36].

Beyond its established biological role in epigenetic regulation, EZH2 has emerged as a promising chromatin-associated biomarker in squamous malignancies. Elevated EZH2 expression has been associated with aggressive clinicopathological features and may help identify patients at increased risk of disease progression. Its dual relevance as both a biomarker and a potential therapeutic target makes EZH2 particularly attractive within biomarker-guided management strategies. Recent evidence further supports its potential utility in the identification of high-risk LSCC subsets, although additional validation in larger disease-specific cohorts remains necessary [40].

Importantly, the future clinical value of molecular biomarkers will likely depend not on individual markers alone but on the integration of multiple biological dimensions. Combining genetic, epigenetic, immune-related, and microenvironment-associated biomarkers may provide a more comprehensive representation of disease biology and improve the precision of risk stratification models.

Collectively, molecular and epigenetic biomarkers represent promising tools for complementing conventional clinicopathological assessment and advancing precision oncology in LSCC. Future studies specifically focused on LSCC are needed to validate candidate biomarkers, define their clinical utility, and integrate them into multidimensional diagnostic and prognostic frameworks capable of supporting personalized patient management.

### 4.2. Liquid Biopsy Biomarkers

Liquid-biopsy approaches have recently gained considerable interest owing to their minimally invasive nature and potential for dynamic disease monitoring. Cell-free DNA (cfDNA), circulating tumor-derived nucleic acids, and other blood-based biomarkers may provide clinically relevant information regarding tumor burden, molecular alterations, treatment response, minimal residual disease, and recurrence risk. Emerging evidence suggests that cfDNA-based approaches may become valuable components of future precision oncology strategies for squamous cell carcinomas of the oral cavity, including LSCC [20].

Beyond cfDNA, circulating non-coding RNAs, extracellular vesicles, and composite molecular signatures are being actively investigated as potential tools for early diagnosis, prognostic assessment, and longitudinal monitoring. Owing to their accessibility through minimally invasive sampling procedures, these biomarkers offer the possibility of repeatedly evaluating tumor-associated biological changes throughout the disease course [19,20].

A major advantage of liquid-biopsy approaches lies in their ability to capture the dynamic and heterogeneous nature of tumor evolution. Repeated sampling may allow real-time assessment of molecular alterations, clonal evolution, treatment response, and emerging resistance mechanisms, thereby providing insights that are difficult to obtain through conventional tissue biopsies alone. In this regard, liquid biopsy has the potential to complement tissue-based analyses and enhance personalized disease monitoring.

Importantly, within the conceptual framework proposed in this review, liquid-biopsy biomarkers may offer a unique opportunity to integrate information derived from multiple biological dimensions. Rather than reflecting a single molecular event, circulating biomarkers may capture the cumulative effects of genetic and epigenetic alterations, immune responses, TME remodeling, and host-related biological changes. As a result, liquid-biopsy approaches could facilitate the development of multidimensional biomarker signatures more closely aligned with the biological complexity of LSCC.

Although most liquid-biopsy applications remain in the validation phase and LSCC-specific evidence is still limited, these technologies hold considerable promise for improving disease surveillance, risk stratification, early detection of recurrence, and treatment monitoring. Future studies integrating circulating biomarkers with molecular, immune, and microenvironment-associated parameters may further enhance the implementation of precision oncology strategies in LSCC.

### 4.3. Immune and Microbiome-Related Biomarkers

Increasing attention has focused on biomarkers derived from the TME and immune landscape. Alterations in immune-cell populations, particularly Langerhans cells, have been associated with disease progression and metastatic behavior, supporting their potential utility for disease assessment and patient stratification [10].

Beyond their biological role in carcinogenesis, immune-related signatures are increasingly being investigated for their potential clinical relevance. In particular, biomarkers associated with immune surveillance, immune checkpoint activation, and stromal–immune interactions may provide additional information regarding disease aggressiveness and treatment responsiveness [6,11].

Similarly, CAF-related signatures have emerged as promising indicators of tumor behavior, reflecting the growing recognition that stromal components actively shape disease progression and therapeutic responsiveness [6,11].

The oral microbiome also represents a promising source of future biomarkers. Microbiome-associated signatures and inflammatory profiles have demonstrated potential utility for early detection and risk stratification. Furthermore, advances in oral barrier immunometabolism research suggest that integrated host–microbiome–immune biomarkers may provide novel opportunities for personalized disease assessment and monitoring [12,14,15].

Collectively, these observations support the concept that immune- and microbiome-related biomarkers may complement conventional molecular markers and contribute to a more comprehensive characterization of disease biology, patient stratification, and future precision oncology approaches.

### 4.4. AI and Integrative Predictive Models

Artificial intelligence (AI) and machine-learning approaches are increasingly being incorporated into biomarker discovery and translational cancer research. By integrating molecular, histopathological, radiological, clinical, and increasingly microenvironment-related data, these technologies offer unprecedented opportunities to identify complex biological patterns that may not be readily detectable using conventional analytical methods [19].

One of the major strengths of AI-based approaches lies in their ability to manage and interpret large-scale, multidimensional datasets. As the molecular characterization of squamous malignancies continues to expand, computational tools are becoming increasingly valuable for integrating information derived from genetic and epigenetic alterations, signaling pathways, immune profiles, liquid-biopsy biomarkers, microbiome-associated signatures, and clinical variables into unified predictive models [18,19].

In addition, AI-driven predictive systems have demonstrated promising potential in enhancing diagnostic accuracy, risk stratification, prognostic assessment, and treatment selection. In the specific context of LSCC, recent studies have explored AI-based approaches for predicting cervical lymph node metastasis and supporting preoperative clinical decision-making, highlighting their potential translational value [18,19].

Importantly, within the conceptual framework proposed in this review, AI may serve not simply as a data-analysis tool but as an enabling technology capable of integrating the multiple biological dimensions that contribute to LSCC pathogenesis. By combining molecular, immune, microbiome-related, stromal, radiological, and clinicopathological information, AI-based models may facilitate the identification of multidimensional biomarker signatures that more accurately reflect disease complexity and individual patient risk.

Future developments are likely to focus on the construction of integrative predictive models capable of capturing dynamic interactions among UV-induced molecular alterations, immune regulation, microbiome-associated mechanisms, and TME remodeling. Such approaches may ultimately support more precise patient stratification, personalized surveillance strategies, and biologically informed therapeutic decision-making. Although these technologies remain largely investigational in LSCC, their ability to integrate heterogeneous data sources positions them as a key component of future precision oncology frameworks. As larger, well-annotated datasets become available, AI-driven approaches may play an increasingly important role in translating complex biological information into clinically actionable decision-support tools. The principal categories of emerging biomarkers currently under investigation in LSCC, together with their potential clinical applications, are summarized in Table 2.

Despite these promising developments, most candidate biomarkers remain in the exploratory or validation phase, and direct evidence specifically focused on LSCC is still limited. Additional multicenter studies, prospective clinical validation, and standardized analytical pipelines will be required to establish clinical utility, reproducibility, and cost-effectiveness before routine implementation can be achieved.

Overall, the integration of molecular, epigenetic, immune-related, microbiome-associated, stromal, and liquid-biopsy biomarkers has the potential to transform LSCC management. Within the conceptual framework proposed in this review, future precision medicine approaches will likely combine conventional clinicopathological assessment with multidimensional biomarker profiling and AI-driven predictive models, facilitating earlier diagnosis, improved patient stratification, personalized therapeutic decision-making, and more precise disease monitoring.

## 5. Translational and Therapeutic Implications

Recent advances in the understanding of LSCC biology have identified a range of molecular, immunological, and microenvironmental mechanisms that may represent promising targets for future therapeutic intervention. As the biological complexity of LSCC becomes increasingly apparent, there is growing recognition that effective therapeutic strategies will likely require approaches capable of addressing multiple interconnected drivers of disease rather than isolated pathogenic mechanisms.

Dysregulation of signaling pathways such as EGFR, PI3K/AKT/mTOR, MAPK, NF-κB, and STAT3 has been implicated in tumor progression and represents a potential source of therapeutic vulnerabilities. These pathways regulate key biological processes, including proliferation, survival, immune modulation, metabolic adaptation, and resistance to therapy. While direct evidence in LSCC remains limited, studies performed in OSCC and other squamous cell carcinomas support the potential relevance of pathway-directed therapeutic approaches in selected molecular contexts [4,6].

Increasing attention has also focused on immune-related mechanisms. The progressive establishment of an immunosuppressive TME, characterized by impaired immune surveillance, immune checkpoint activation, stromal remodeling, and immune-cell dysfunction, suggests that immunomodulatory strategies may represent an important avenue for future investigation. In particular, the PD-1/PD-L1 axis, immune-cell composition, and stromal–immune interactions have emerged as potential targets for both therapeutic intervention and biomarker-guided patient selection [5,6,11].

The growing recognition of the role of CAFs and stromal signaling further expands the spectrum of potential therapeutic opportunities. CAF-mediated pathways involved in extracellular matrix remodeling, immune regulation, angiogenesis, and treatment resistance may represent promising targets for future combination strategies aimed at modifying the TME and improving therapeutic responsiveness [6,11].

Similarly, increasing interest has been directed toward the microbiome–immune–metabolic axis as a potential therapeutic target. Emerging evidence suggests that host–microbiome interactions may influence inflammation, immune regulation, epithelial homeostasis, and treatment responsiveness. Although these approaches remain largely investigational, microbiome-associated mechanisms may provide novel opportunities for preventive, prognostic, and therapeutic interventions. These concepts are discussed further in the following section [14,15].

Importantly, the translational significance of these discoveries lies not only in the identification of individual therapeutic targets but also in their integration within a broader biological framework. Molecular alterations, immune responses, stromal remodeling, and microbiome-associated pathways are increasingly recognized as interconnected components of a dynamic disease network. Consequently, future therapeutic strategies may benefit from combination approaches capable of simultaneously targeting multiple biological dimensions of LSCC.

Collectively, these findings support a progressive transition from conventional clinicopathological management toward biologically informed and biomarker-guided models of care. Within the conceptual framework proposed in this review, future precision oncology strategies will likely integrate molecular profiling, immune characterization, microenvironmental assessment, and microbiome-associated signatures to improve patient stratification, optimize therapeutic decision-making, and facilitate the development of personalized treatment approaches.

### Microbiome-Targeted Therapeutic Strategies

**Figure 6:**
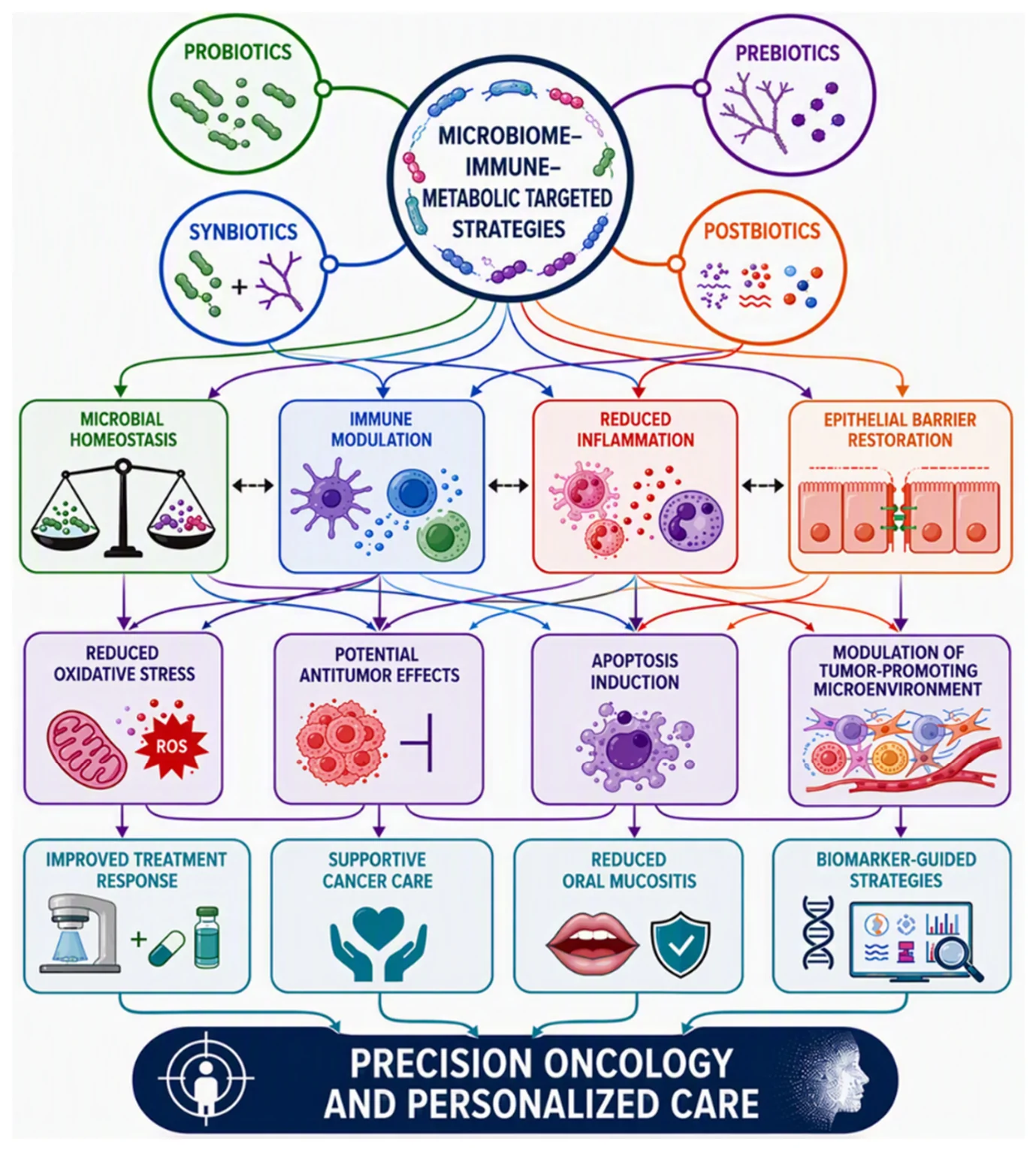
Microbiome-targeted therapeutic strategies in LSCC. Probiotics, prebiotics, synbiotics, and postbiotics may modulate the microbiome–immune–metabolic axis by promoting microbial homeostasis, restoring epithelial barrier integrity, regulating inflammatory responses, and enhancing immune function. Through these interconnected mechanisms, microbiome-targeted interventions may influence oxidative stress, tumor-promoting microenvironmental conditions, treatment-related toxicity, and therapeutic responsiveness. Although their role in LSCC remains largely investigational, these approaches represent a promising translational extension of the microbiome–immune–metabolic framework and may contribute to future biomarker-guided precision oncology strategies. Colors are used to distinguish different microbiome-targeted intervention, biological processes, and clinical outcomes, while arrows indicate the direction of interactions and downstream effects. Symbols represent key molecular, cellular, and therapeutic features associated with microbiome-based strategies in LSCC.

Recent advances in oral microbiome research have stimulated growing interest in microbiome-targeted interventions as potential complementary approaches for cancer prevention and management (Figure 6).

Among these approaches, probiotics, prebiotics, synbiotics, and postbiotics have emerged as promising candidates for modulation of the oral microbiome and its associated biological functions. Collectively, these interventions may influence microbial composition, metabolite production, epithelial barrier integrity, inflammatory responses, and host immune regulation, thereby counteracting several mechanisms implicated in oral carcinogenesis [16].

Probiotics have been the most extensively investigated microbiome-based intervention in oral cancer research. Proposed mechanisms include inhibition of pathogenic microbial colonization, modulation of immune responses, attenuation of chronic inflammation, reduction in oxidative stress, enhancement of epithelial barrier function, and regulation of tumor-associated signaling pathways [16,44]. Experimental studies have additionally reported direct antitumor effects of selected probiotic strains, including growth inhibition, modulation of cellular proliferation, and induction of apoptosis in OSCC models [45,46].

Accumulating evidence further suggests that microbiome-targeted interventions may influence several molecular pathways involved in oral carcinogenesis. Dysbiosis-associated microorganisms have been linked to activation of NF-κB and STAT3 signaling, production of ROS, modulation of host immune responses, interference with tumor suppressor pathways, and enhancement of invasive tumor phenotypes. Consequently, restoration of microbial balance has been proposed as a potential strategy to mitigate tumor-promoting biological processes and support host immune function [17].

Prebiotics, defined as substrates selectively utilized by beneficial microorganisms, may indirectly influence host biology by promoting the growth and activity of health-associated microbial communities and supporting the production of bioactive metabolites with immunomodulatory and anti-inflammatory properties. Synbiotics, combining probiotics with prebiotic substrates, have been proposed as a strategy to enhance microbial engraftment and maximize biological effects [16].

More recently, increasing attention has focused on postbiotics, including microbial metabolites, cell-wall components, and other bioactive products derived from beneficial microorganisms. Unlike live microorganisms, postbiotics may offer greater formulation stability and a potentially improved safety profile while retaining anti-inflammatory, immunomodulatory, and microbiome-regulatory properties. These characteristics have generated growing interest in their potential translational applications in oral cancer prevention and management [16].

Beyond potential anticancer effects, microbiome-targeted interventions may also contribute to supportive cancer care. A systematic review and meta-analysis reported that probiotic supplementation may reduce the severity of treatment-associated oral mucositis, suggesting a beneficial role in improving treatment tolerance and oral health outcomes among oncology patients [47]. Furthermore, a recent randomized clinical trial in patients with head and neck cancer demonstrated favorable effects of probiotic supplementation on oral clinical and microbiological parameters, supporting the feasibility of oral microbiome modulation in clinical settings [48].

Despite these promising observations, current evidence remains insufficient to support routine implementation of probiotics, prebiotics, synbiotics, or postbiotics as standard approaches for LSCC prevention or treatment. Most published data derive from experimental models or broader oral cancer populations, whereas LSCC-specific clinical evidence remains limited [16,44]. Consequently, additional mechanistic investigations and well-designed clinical studies are required before microbiome-targeted interventions can be incorporated into evidence-based management strategies.

Overall, microbiome-targeted therapeutic strategies may represent a promising translational extension of the microbiome–immune–metabolic axis concept. Beyond conventional probiotic supplementation, emerging concepts such as microbiome-targeted immunomodulators, engineered bacteriotherapy, and microbiota-based diagnostics further expand the spectrum of potential translational applications, although their role in LSCC remains largely investigational [17]. Future research may clarify whether restoration of microbial homeostasis can contribute to disease prevention, enhance treatment responsiveness, and complement emerging biomarker-guided precision oncology approaches in LSCC.

## 6. Discussion

The evidence summarized in this review reflects a substantial evolution in the current understanding of LSCC. Traditionally regarded as a predominantly UV-driven malignancy, LSCC is increasingly recognized as a biologically heterogeneous disease arising from the interaction of environmental exposures, molecular alterations, immune regulation, host–microbiome dynamics, and TME remodeling.

One of the most significant conceptual advances emerging from recent research is the transition from a predominantly tumor-centered model of carcinogenesis toward a more integrated biological framework. Although UV-induced DNA damage remains the initiating event in most cases, disease progression appears to be shaped by extensive crosstalk among genetic and epigenetic alterations, dysregulated signaling pathways, inflammatory networks, immune surveillance mechanisms, and stromal interactions. This integrated perspective provides a more comprehensive explanation for the substantial biological variability observed among patients with apparently similar clinicopathological characteristics.

Particularly noteworthy is the increasing recognition of immunopathological mechanisms as central contributors to disease evolution. The growing appreciation of immune surveillance dysfunction, immune checkpoint signaling, and stromal–immune interactions highlights the importance of the local biological ecosystem in determining disease progression and therapeutic responsiveness. In this context, the TME is increasingly regarded as an active driver of carcinogenesis rather than a passive consequence of malignant transformation.

Another major conceptual development concerns the recognition of host–microbiome interactions as biologically relevant modulators of carcinogenesis. The microbiome–immune–metabolic axis provides a useful framework for understanding how microbial communities may influence inflammation, immune regulation, epithelial homeostasis, and disease progression. In parallel, the possibility of modulating microbial ecosystems to influence these processes has introduced a promising translational dimension to contemporary oral cancer research.

Despite these advances, important limitations remain. A substantial proportion of the available molecular evidence derives from OSCC or cSCC, whereas LSCC-specific mechanistic studies remain comparatively limited. Consequently, caution is warranted when extrapolating biological findings from related tumor entities to LSCC. Furthermore, many proposed biomarkers and molecular targets remain supported primarily by exploratory or proof-of-concept studies and require validation in adequately powered prospective cohorts.

The growing interest in molecular, epigenetic, immune-related, microbiome-associated, and liquid-biopsy biomarkers represents one of the most promising areas of contemporary LSCC research. Likewise, AI-based analytical tools offer new opportunities for integrating heterogeneous clinical and biological datasets into multidimensional predictive models. However, successful translation of these advances into routine clinical practice will require methodological standardization, multicenter validation, and demonstration of clinical utility and cost-effectiveness.

Overall, current evidence supports a multidimensional interpretation of LSCC in which environmental exposure, molecular alterations, immune regulation, stromal interactions, and microbial ecosystems collectively contribute to disease behavior. Continued integration of these complementary perspectives will be essential for advancing both the biological understanding of LSCC and the development of future biomarker-driven and biologically informed management strategies.

## 7. Conclusions

LSCC is increasingly recognized as a biologically complex malignancy whose development and progression cannot be explained solely by chronic UV exposure. Although UV-induced photodamage remains the principal initiating event, contemporary evidence indicates that carcinogenesis results from dynamic interactions among genetic and epigenetic alterations, dysregulated signaling pathways, immune responses, TME remodeling, chronic inflammation, and microbiome-associated mechanisms.

Recent advances have expanded the traditional view of LSCC, highlighting the important contribution of immunopathological processes, stromal–immune crosstalk, CAFs, oral dysbiosis, and disruption of the microbiome–immune–metabolic axis. Together, these mechanisms contribute to disease heterogeneity and help explain differences in tumor behavior, progression, and therapeutic responsiveness.

The identification of emerging biomarkers, including non-coding RNAs, epigenetic signatures, liquid-biopsy analytes, immune-related markers, microbiome-associated profiles, and integrative computational models, is creating new opportunities for patient stratification and precision oncology. Although most of these approaches remain investigational, they provide a strong rationale for the development of multidimensional biomarker frameworks capable of complementing conventional clinicopathological assessment.

Future research should prioritize LSCC-specific molecular investigations, multicenter biomarker-validation studies, and integrated models incorporating molecular, immunological, microbiome-related, and clinical variables. Such efforts may facilitate the implementation of biomarker-guided and biologically informed management strategies, ultimately improving our understanding of LSCC, supporting the transition toward more personalized approaches to cancer care, and informing the development of microbiome-targeted therapeutic strategies.

## Figures and Tables

**Table 1:** Comparative overview of major biological and clinical characteristics of LSCC, OSCC, and cSCC.

Feature	LSCC	OSCC	cSCC	Key References
Principal anatomical site	Lip vermilion and mucocutaneous junction	Oral mucosa	Skin	[1]
Major risk factors	UV exposure; tobacco/alcohol	Tobacco, alcohol, betel quid; HPV in selected cases	UV exposure; immunosuppression	[1,27]
Biological environment	Oral–cutaneous interface	Oral mucosal environment	Cutaneous environment	[1]
Predominant carcinogenic driver	UV-induced photodamage	Chemical carcinogens and chronic inflammation	UV-induced photodamage	[4,8]
UV-signature mutations	Characteristic	Uncommon	Characteristic	[7,8]
*TP53* alterations	Frequent	Frequent	Frequent	[3,7]
TME evidence	Emerging	Well-characterized	Well-characterized	[5,6]
Oral microbiome involvement	Emerging evidence	Increasingly characterized	Limited evidence	[12,14]
Biomarker development	Limited LSCC-specific data	Extensive	Expanding evidence base	[19,20]
Precision oncology	Emerging	Increasingly implemented	Increasingly implemented	[6,19]
Evidence base	Limited	Extensive	Extensive	[1,4]

**Table 2:** Emerging molecular, epigenetic, immune-, microbiome-, and liquid biopsy-related biomarkers with potential diagnostic, risk-stratification, and therapeutic relevance in LSCC.

Biomarker Category	Representative Biomarkers	Potential Clinical Application	Key References
miRNAs	miR-21, miR-155, miR-375	Diagnosis, monitoring	[37]
lncRNAs	H19, HOTAIR, MALAT1, ANRIL	Prognostic biomarkers; therapeutic targets	[38,39]
Epigenetic biomarkers	DNA methylation signatures, histone modifications	Early detection; risk stratification	[9,36]
Chromatin regulators	EZH2	Risk stratification; target identification	[40]
Liquid biopsy biomarkers	cfDNA, circulating tumor-derived nucleic acids	Disease monitoring; Recurrence surveillance	[20]
Immune biomarkers	Langerhans cells, immune cell signatures	Immune profiling; risk stratification	[10]
TME biomarkers	CAF signatures, PD-1/PD-L1	Treatment selection	[6,11]
Microbiome-related biomarkers	Microbial inflammatory profiles	Early detection; risk stratification	[12,14,41]
Integrated host–microbiome biomarkers	Immunometabolic signatures	Precision assessment	[15]
AI-assisted biomarker models	Multi-omics predictive models	Precision diagnosis; risk prediction	[18,19]

## Data Availability

No new data were created or analyzed in this study. Data sharing is not applicable to this article.

## Abbreviations

The following abbreviations are used in this manuscript:

6–4PPs	Pyrimidine-(6–4)-pyrimidone photoproducts
AI	Artificial intelligence
AKT	Protein kinase B
CAFs	Cancer-associated fibroblasts
cfDNA	Cell-free DNA
CPDs	Cyclobutane pyrimidine dimers
cSCC	Cutaneous squamous cell carcinoma
CXCL12	C-X-C motif chemokine ligand 12
CXCR4	C-X-C chemokine receptor type 4
ECM	Extracellular matrix
EGFR	Epidermal growth factor receptor
EZH2	Enhancer of Zeste Homolog 2
H3K27me3	Histone H3 lysine 27 trimethylation
IL-1β	Interleukin-1 Beta
IL-6	Interleukin-6
lncRNAs	Long non-coding RNAs
LSCC	Lip squamous cell carcinoma
MAPK	Mitogen-activated protein kinase
MDSCs	Myeloid-derived suppressor cells
miRNAs	MicroRNAs
mTOR	Mammalian target of rapamycin
NER	Nucleotide excision repair
NF-κB	Nuclear factor kappa B
OSCC	Oral squamous cell carcinoma
PD-1	Programmed cell death protein 1
PD-L1	Programmed death-ligand 1
PI3K	Phosphoinositide 3-kinase
PRC2	Polycomb repressive complex 2
ROS	Reactive oxygen species
STAT3	Signal transducer and activator of transcription 3
TAMs	Tumor-associated macrophages
TME	Tumor microenvironment
TNF-α	Tumor necrosis factor-alpha
*TP53*	Tumor protein p53
Tregs	Regulatory T cells
UV	Ultraviolet

## References

[1] Howard A., Agrawal N., Gooi Z. (2021). Lip and Oral Cavity Squamous Cell Carcinoma. Hematol. Oncol. Clin. N. Am..

[2] Ghanem A.S., Toth A., Nagy A.C. (2025). Socio-demographic disparities in global trends of lip and oral cavity neoplasms from 1990 to 2021. Sci. Rep..

[3] Leemans C.R., Snijders P.J.F., Brakenhoff R.H. (2018). The molecular landscape of head and neck cancer. Nat. Rev. Cancer.

[4] Johnson D.E., Burtness B., Leemans C.R., Lui V.W.Y., Bauman J.E., Grandis J.R. (2020). Head and neck squamous cell carcinoma. Nat. Rev. Dis. Primers.

[5] Li C., Dong X., Li B. (2024). Tumor microenvironment in oral squamous cell carcinoma. Front. Immunol..

[6] Tsai C.C., Hsu P.C., Kuo C.Y. (2026). Molecular Mechanisms in Oral Squamous Cell Carcinoma: Integrative Roles of Cancer-Associated Fibroblasts, Immune Microenvironment, and Precision Therapeutic Opportunities. Int. J. Mol. Sci..

[7] Brash D.R., Simon J.A., Lin A., McKenna G.J., Baden H.P., Halperin A.J., Pontén J. (1991). A role for sunlight in skin cancer: UV-induced p53 mutations in squamous cell carcinoma. Proc. Natl. Acad. Sci. USA.

[8] Brash D.E. (2015). UV signature mutations. Photochem. Photobiol..

[9] Mesgari H., Esmaelian S., Nasiri K., Ghasemzadeh S., Doroudgar P., Payandeh Z. (2023). Epigenetic Regulation in Oral Squamous Cell Carcinoma Microenvironment: A Comprehensive Review. Cancers.

[10] Marega N.A., da Costa E.S.L.P.B., de Oliveira A.B., de Carli R.L.B., Bufalino A., Massucato E.M.S., Ferrisse T.M. (2025). Different densities of Langerhans cells among oral squamous cell carcinoma, lip squamous cell carcinoma and oral potentially malignant disorders: Systematic review and network meta-analysis. Arch. Oral Biol..

[11] Liu X., Wang D., Cheng X., Ma Y., Li S., Deng J., Qiao C. (2025). Fibroblast-immune crosstalk in oral squamous cell carcinoma: From tumor promotion to immune evasion. Front. Immunol..

[12] Peter T.K., Withanage M.H.H., Comnick C.L., Pendleton C., Dabdoub S., Ganesan S., Drake D., Banas J., Xie X.J., Zeng E. (2022). Systematic review and meta-analysis of oral squamous cell carcinoma associated oral microbiome. Front. Microbiol..

[13] Dumitrescu R., Bolchis V., Fratila A.D., Jumanca D., Buzatu B.L.R., Sava-Rosianu R., Alexa V.T., Galuscan A., Balean O. (2025). The Global Trends and Advances in Oral Microbiome Research on Oral Squamous Cell Carcinoma: A Systematic Review. Microorganisms.

[14] Santacroce L., Jirillo E., Bottalico L., Topi S., Passarelli P.C., Mancini A., D’Addona A., Garcia Godoy F., Folliero V., Colella M. (2025). Does a link exist between oral microbiota and oral squamous cell carcinoma? A review of current insights. J. Oral Microbiol..

[15] Ademi A., Topi S., Gugu M., Ciafarone A., Cifone M.G., Pietropaoli D., Altamura S. (2026). Oral Barrier Immunometabolism in Chronic Low-Grade Inflammation: Molecular Mechanisms and Systemic Implications. Int. J. Mol. Sci..

[16] Mohd Fuad A.S., Amran N.A., Nasruddin N.S., Burhanudin N.A., Dashper S., Arzmi M.H. (2023). The Mechanisms of Probiotics, Prebiotics, Synbiotics, and Postbiotics in Oral Cancer Management. Probiotics Antimicrob. Proteins.

[17] Urzica R.N., Cretu B., Caruntu A., Bucurica S., Farcasiu A.T., Ciupescu L.M., Scheau C., Caruntu C. (2025). The Molecular Interplay Between Oral Microbiome and Oral Cancer Pathogenesis. Int. J. Mol. Sci..

[18] Senirli R.T., Simsek M.A., Kocak E., Sonbay Yilmaz N.D., Selcuk O.T. (2026). AI-based Prediction of Neck Metastasis in Early-stage Lip Squamous Cell Carcinoma Using Preoperative Clinical-morphologic Features. J. Craniofacial Surg..

[19] Ardila C.M., Vivares-Builes A.M., Pineda-Velez E. (2026). Molecular Biomarkers and Machine Learning in Oral Cancer: A Systematic Review and Meta-Analysis. Oral Dis..

[20] Veiga P., Barroso L., Pires L.M., Mano C., Caramelo F., Carreira I.M., Ribeiro I.P., de Melo J.B. (2026). Cell-Free DNA as Biomarker in Oral Squamous Cell Carcinoma: Dynamics, Mutational Landscape and Clinical Implications. Cells.

[21] Rikitake R., Mizushima Y., Yamamoto Y., Higashi T., Sakai T., Kawai A., Asakage T. (2026). Subsite- and histology-specific epidemiology of head and neck cancers in Japan from 2016 to 2019: An application of a new classification of rare cancers. Int. J. Clin. Oncol..

[22] Rumgay H., Colombet M., Ramos da Cunha A., Filho A.M., Warnakulasuriya S., Conway D.I., Chaturvedi A., Virani S., Lauby-Secretan B., Carvalho A.L. (2026). Global incidence of lip, oral cavity, and pharyngeal cancers by subsite in 2022. CA Cancer J. Clin..

[23] Koyama S., Morishima T., Nakata K., Nishimura N., Ishibashi M., Miyashiro I. (2026). Oral Squamous Cell Carcinoma Incidence in Japan Based on National Cancer Registry Data 2016–2019. JMA J..

[24] Warnakulasuriya S. (2009). Global epidemiology of oral and oropharyngeal cancer. Oral Oncol..

[25] Girardi F.M., de Farias Gabriel A., Wagner V.P., Martins M.D. (2025). Second Events After Curative-Intent Treatment for Lip Cancer: Navigating the Path of Follow-Up Care. Oral Dis..

[26] Toprani S.M., Kelkar Mane V. (2021). A short review on DNA damage and repair effects in lip cancer. Hematol. Oncol. Stem Cell Ther..

[27] Coletta R.D., Yeudall W.A., Salo T. (2024). Current trends on prevalence, risk factors and prevention of oral cancer. Front. Oral Health.

[28] Kannan N., Ramalingam K., Kizhakkoottu S., Ramani P. (2024). The Diagnostic Considerations and Clinical Management of Lower Lip Swellings in Adolescents: A Narrative Review. Cureus.

[29] Carneiro M.C., Quenta-Huayhua M.G., Peralta-Mamani M., Honorio H.M., Santos P., Rubira-Bullen I.R.F., Rubira C.M.F. (2023). Clinicopathological Analysis of Actinic Cheilitis: A Systematic Review with Meta-analyses. Head. Neck Pathol..

[30] Obermeier K.T., Bachmann E., Dewenter I., Malenova Y., Poxleitner P., Liokatis P., Smolka W. (2026). Understanding the risk of metastasis in lower lip carcinoma: Clinical insights and prognostic implications. Head. Face Med..

[31] Milano A.F. (2023). Lip Cancer: 20-Year Comparative Survival and Mortality Analysis by Age, Sex, Race, Stage, Grade, Cohort Entry Time-Period and Disease Duration A Systematic Review of 19,213 Cases for Diagnosis Years 1973-2014: (SEER*Stat 8.3.5). J. Insur. Med..

[32] Hafstrom A., Hammerlid E., Beran M., Olin M., Hogmo A., Farnebo L. (2026). Treatment and outcome for 2,111 patients with vermilion lip squamous cell carcinoma: A nationwide, population-based study from the SweHNCR. Oral Oncol..

[33] Pfeifer G.P., Besaratinia A. (2012). UV wavelength-dependent DNA damage and human non-melanoma and melanoma skin cancer. Photochem. Photobiol. Sci..

[34] Cadet J., Douki T. (2018). Formation of UV-induced DNA damage contributing to skin cancer development. Photochem. Photobiol. Sci..

[35] Norval M., Halliday G.M. (2011). The consequences of UV-induced immunosuppression for human health. Photochem. Photobiol..

[36] Marmo G.M., Munzone M., Polizzi A., Campagna R., Mascitti M., Isola G. (2025). Genetic and Epigenetic Biomarkers for the Early Oral Cancerization Risk in Periodontitis Patients. Curr. Issues Mol. Biol..

[37] Abdul N.S., Shenoy M., Shivakumar G.C., Patel S., Jyoti B., Shivakumar S. (2025). Evaluation of microRNA Expression Profiles as Diagnostic and Prognostic Biomarkers in Oral Squamous Cell Carcinoma: A Molecular Approach. J. Pharm. Bioallied Sci..

[38] Rajendran P., Sekar R., Abdallah B.M., Jh S.F., Ali E.M., Jayaraman S., Abdelsalam S.A., Veeraraghavan V. (2024). Epigenetic modulation of long noncoding RNA H19 in oral squamous cell carcinoma-A narrative review. Noncoding RNA Res..

[39] Bhattacharyya R., Biswas C., Khan M.S., Dutta S., Sur S. (2026). Circulating lncRNA as Biomarkers and Therapeutics in Oral Cancer: Silent Scripts with Loud Impact. Mol. Biol. Rep..

[40] Valea C.V., Klein M., Hallermann C., Schulze H.J., Raguse J.D., Wermker K. (2024). EZH2: An analysis of a potential new tumor marker in high-risk localization of cutaneous squamous cell carcinomas. Front. Oncol..

[41] Cao Q., Yang Y., Bai S., Shen J. (2026). The oral bacterial microbiota change in oral squamous cell carcinoma: A systematic review and meta-analysis. BMC Oral Health.

[42] Yu X., Shi Y., Yuan R., Chen Z., Dong Q., Han L., Wang L., Zhou J. (2023). Microbial dysbiosis in oral squamous cell carcinoma: A systematic review and meta-analysis. Heliyon.

[43] Menghani S.V. (2025). Carcinogenetic mechanisms employed by the oral microbiome: A narrative review. Am. J. Med. Sci..

[44] Rivera C. (2025). Understanding the potential of probiotics in oral cancer prevention: A short review on microbial modulation. Discov. Oncol..

[45] Nami Y., Tavallaei O., Kiani A., Moazami N., Samari M., Derakhshankhah H., Jaymand M., Haghshenas B. (2024). Anti-oral cancer properties of potential probiotic lactobacilli isolated from traditional milk, cheese, and yogurt. Sci. Rep..

[46] Rehaman T.A., Bhardwaj R.G., Sharma N., Ellepola A., Karched M. (2026). Probiotic Lactobacillus casei Inhibits Oral Squamous Cell Carcinoma Growth and Induces Apoptosis In Vitro. Int. Dent. J..

[47] Liu Y.C., Wu C.R., Huang T.W. (2022). Preventive Effect of Probiotics on Oral Mucositis Induced by Cancer Treatment: A Systematic Review and Meta-Analysis. Int. J. Mol. Sci..

[48] Pereira Riveros T., Jane Salas E., Lozano Borbalas A., Aguilera F.R., Vinuesa Aumedes T. (2025). Effect of a Probiotic Combination on Clinical and Microbiological Oral Parameters in Head and Neck Cancer Patients: A Randomised Clinical Trial. Cancers.

